# Burden of Disease Associated with Dietary Exposure to Aflatoxins in China in 2020

**DOI:** 10.3390/nu14051027

**Published:** 2022-02-28

**Authors:** Tingting Chen, Jialin Liu, Yiling Li, Sheng Wei

**Affiliations:** School of Public Health, Tongji Medical College, Huazhong University of Science and Technology, Wuhan 430030, China; d202081549@hust.edu.cn (T.C.); m202075429@hust.edu.cn (J.L.); m202075431@hust.edu.cn (Y.L.)

**Keywords:** aflatoxin, burden of disease, risk assessment, dietary exposure, cancer risk

## Abstract

Aflatoxins (AFTs), as a group 1 carcinogen, could lead to hepatocellular carcinoma (HCC). Dietary intake is the primary way of AFT exposure in humans. However, the contribution of foodborne AFT intake to the HCC burden remains unknown in recent years in China. Hence, the present study was conducted to estimate the burden of HCC attributed to foodborne AFT exposure by using disability-adjusted life years (DALYs). The risk assessment was used to estimate the incidence of HCC related to AFT exposure. Concentrations of AFTs in peanuts, peanut oil, corn, and corn products were retrieved from literature published between 2010 and 2020 in China. Corresponding daily food consumption data were obtained from two nationwide Chinese surveys. A direct approach was used to calculate DALY and DALY rates to quantify the HCC burden attributed to dietary AFT exposure. The total amount of AFT intake through peanut, peanut oil, corn, and corn products was 4.018 ng/kg bw/day resulting in 0.125 extra HCC cases per year/100,000 persons, corresponding to a DALY number and DALY rate of 21,625.08 and 1.53 per 100,000 population, respectively. Regionally, DALYs were high in Guangxi and Guangdong provinces, corresponding to 5948 and 5595 DALYs. A total of 1.5 DALYs/100,000 were lost due to the AFT exposure. DALYs per 100,000 population were higher in several coastal areas. Though the disease burden of HCC caused by dietary AFTs was low in the Chinese population, a high health risk was found in the residents of some areas with high AFT exposure. AFTs are still a health challenge for the Chinese people.

## 1. Introduction

Aflatoxins (AFTs) are secondary metabolites produced by *Aspergillus flavus* and *Aspergillus parasiticus* [1]. The most prominent four types of AFTs are aflatoxins B1, B2, G1, and G2 [2]. Among them, AFB1 is the most common and toxic type. AFTs are classified as a group 1 carcinogen by the International Agency for Research on Cancer (IARC) due to their hepatotoxicity, carcinogenicity, and genotoxicity [3]. Researchers also observed that chronic exposure to aflatoxins reduces immunity and interferes with protein metabolism [4,5]. AFTs can be detected in various food, such as cereals, legumes, oilseeds, nuts, spices, and milk, and marine products are also contaminants by AFTs [6]. Peanut and corn are the most common daily foods that are susceptible to contamination by AFTs [7]. Human biomonitoring also confirmed that humans are exposed to aflatoxins [8]. Although AFTs are harmful to humans, achieving zero exposure is difficult because they are often present in commonly consumed food. Due to the toxicity of AFTs, many countries have established standards for restricting aflatoxins. The European Commission has set the maximum levels (MLs) of AFB1 and AFTs to be 2.0 and 4.0 μg/kg in peanuts for direct consumption [9]. According to the current China National Food Safety Standard (GB 2761-2017), the ML of AFB1 is 20 μg/kg in peanut and its products, corn, and corn products [10].

Contamination by AFTs is influenced by environmental and climatic conditions, and the health effects of AFTs are also influenced by demographic characteristics. AFT contamination frequently occurs in tropical and subtropical regions due to the temperature, humidity, soil, and storage conditions [11,12]. The growth rate of aflatoxins is remarkably rapid under high temperature and humid conditions [13]. In China, national monitoring showed that the contamination of AFTs in peanuts and peanut oil was high in several East and South China areas, which have subtropical temperate monsoon climates [14]. In addition to climatic conditions affecting aflatoxin exposure, there are synergistic hepato-carcinogenic effects caused by AFTs and hepatitis B virus (HBV) infection. The cancer potency factor is 0.3 in individuals with chronic HBV infection, while it is 0.01 in individuals without chronic HBV infection [15]. In some developing countries, such as sub-Saharan Africa, which has a high incidence of HCC, chronic exposure to aflatoxin B1 was related to the elevated risk of significant liver fibrosis among chronic HBV carriers [16]. A meta-analysis estimated that AFB1 exposure and HBV infection increased HCC risk by 54 fold [17]. With the increasing rates of administration of the HBV vaccine to newborns in China, the prevalence of HBV has declined quickly from 2000, but at the same time, AFTs may become a more dominant risk for liver cancer in the coming decades due to climate change [18,19]. Therefore, it is necessary to assess the risk of AFTs on the population at current environmental and consumption levels.

The burden of disease is an indicator of the health and economic impact of illness, injury, and early death on societies and countries. Disability adjusted life years (DALYs) is a standard metric to express the burden of disease [20]. Since exposure to contaminants may lead to health loss, DALYs was used to quantify the impact of environmental pollution [21]. Gibb et al. estimated that the number of foodborne illnesses due to aflatoxin was 21,757, and the worldwide foodborne DALYs were 636,869 in 2010 [22]. Liu and Wu estimated the aflatoxin exposure was 17–30 ng/kg body weight/day, and there were 7200 to 18,830 cases of HCC induced by aflatoxin in China and 25,200 to 155,000 cases globally in 2010 [15]. However, little is known regarding the disease burden of HCC caused by foodborne aflatoxin intake in China in the recent decade. Most aflatoxin risk assessment studies mainly focused on AFB1 in a local region or population without disease burden assessment. Based on our knowledge, no published studies reported the risk assessment and disease burden assessment of the dietary exposure to AFTs in peanuts and peanut oil, corn, and corn products from different areas of China. Qin et al. once investigated the spatial distribution of dietary exposure of aflatoxins in peanuts and peanut oil in China; however, they did not assess corn or its products, which are also commonly eaten in China, and did not assess the burden of disease or identify high-risk groups [14].

The present study intended to assess the AFT exposure of the Chinese population by combining consumption data and data on AFT contamination in four kinds of food: peanuts, peanut oil, corn, and corn products. Furthermore, we also estimate the burden of disease of HCC caused by AFT intake through DALY calculation in China. Our findings could provide an updated view on the disease burden for AFT exposure in China.

## 2. Materials and Methods

### 2.1. Study Design

The present study consisted of two parts including the dietary AFT risk assessment and calculation the burden of disease attributed to dietary AFT exposure [15,23]. The risk assessment of dietary AFTs included exposure assessment and risk characterization for AFTs [15]. We obtained the concentrations of AFTs from the literature and combined the consumption data from national surveys to conduct the exposure assessment [24,25]. Then, we described the risk characterization using the exposure data and the dose–response relationship [22]. We estimate the incidence of HCC caused by dietary aflatoxin intake in this section. Finally, the direct method was used to calculate the DALY based on the national cancer registry report and the template recommended by WHO [23]. An overview of the design is shown in Supplementary Figure S1.

### 2.2. Data Collection

A literature review was conducted to obtain the aflatoxins’ concentrations in peanut, peanut oil, corn, and corn products from PubMed and three Chinese databases: The General Library of Chinese Academic Journals (CNKI), Wanfang Data—academic journal full-text library (WAN FANG), and Chinese Biomedical Literature Database (CBM) from the period January 2010 to December 2020.

The keywords for retrieval strategies are shown in Supplementary Table S1. Studies eligible for inclusion met all of the following criteria: (1) the research objects were peanut, peanut oil, corn, or corn products; (2) the sample collection time was from January 2010 to December 2020; (3) detected methods included HPLC, UPLC, and ELISA; (4) concentrations of aflatoxin were described; (5) the study area was in mainland China. A total of 5639 studies were retrieved by the search terms. Among them, 126 studies were full-text reviewed, and 77 papers were removed due to the lack of average data, contaminated area data, or because the time of sampling was not in conformity with our requirements. Finally, we included 47 studies that provided information about the contamination of AFTs in peanut, peanut oil, corn, and corn products. More details on the literature, inclusion criteria, and exclusion criteria are provided in Supplementary Figure S2.

The weighted arithmetic means of aflatoxin concentrations were calculated for the same food types in studies by using the following formula [26]:

$$Ci=\sum_{m=1}^{m=n}\left(N_{m}\times M_{m}\right)/\sum_{m=1}^{m=n}\;N_{m}\;\;$$

where *Ci* is the weighted arithmetic mean of aflatoxin concentrations (μg/kg) in food *i*, $N_{m}$ is the sample size, $M_{m}$ is the mean of the aflatoxin concentration in the original study, and *n* is the number of the studies for each food (peanut, peanut oil, corn, and corn products). For each food, the original concentrations, detection method, and other main information are shown in Supplementary Tables S2–S4.

### 2.3. Consumption Data of Peanut, Peanut Oil, Corn, and Corn Products

The consumption frequency (gram per person per day, g/day) of peanut, peanut oil, corn, and corn products was obtained from two national-level surveys in China. One was the 2010–2012 China Nutrition and Health Surveillance [25], which provided the consumption data of peanut, corn, and corn products from 18 provinces, autonomous regions, and municipalities of mainland China. The other one was the China National Nutrition and Health Survey (CNNHS) of 2002 [24], which provided the consumption data of peanuts from 13 provinces. The consumption data of peanut oil was obtained from the CNNHS of 2002. These surveys were conducted by well-trained dietary staff to obtain three consecutive 24 h dietary recalls via face-to-face interviews (including two weekdays and one weekend day).

### 2.4. Estimation of Daily Intake of Aflatoxins

According to the method recommended by the FAO/WHO [27], the estimated daily foodborne aflatoxin intake by body weight (*EDI*, ng/kg·body weight/day) was calculated using the following formula [28]:

$$EDI_{j}=\frac{C_{ij}\times D_{ij}}{W_{j}}\;$$

where *EDI_j_* (ng/kg body weight/day) is the total daily foodborne aflatoxin intake in *j* province; *C_ij_* is the weighted arithmetic mean of aflatoxin concentrations (μg/kg) in food *i* in *j* province; *D_ij_* is the daily consumption (g/day) of food *i* in *j* province; and *W_j_* is the average body weight (kg) of residents in *j* province.

### 2.5. Risk Characterization

#### 2.5.1. Margin of Exposure (MOE)

The MOE approach, which was proposed by EFSA in 2005, was used to estimate the genotoxic and carcinogenic risk [29]. The MOE was calculated by dividing the benchmark dose lower limit for an additional 10% risk (BMDL_10_) by the human dietary exposure. The value of BMDL_10_ used was 400 ng/kg bw/day as previously derived by EFSA (2020) based on the data on HCC incidences in male rats [30]. MOE values do not quantify the risk but can indicate a level of concern from a public health point of view. An MOE of 10,000 or higher would be considered to indicate a low public health risk. The formula to calculate MOE is as follows:

MOE = BMDL_10_/*EDI*

#### 2.5.2. Risk Assessment of Hepatocellular Carcinoma

We used the population attributable fraction (PAF) approach to estimate the incidence of HCC due to AFTs. A quantitative risk assessment method was used to estimate the excess risk of HCC-related aflatoxins, which was established by JECFA [22]. This assessment was done separately in two populations, with (HBsAg+) and without HBV infection (HBsAg−), because of the synergistic hepato-carcinogenic effects of AFTs and HBV infection. When the dietary exposure of AFTs was 1 ng/kg bw/day, the AFTs’ cancer potency factor in HBsAg− individuals was 0.01 cases/100,000 persons/year, while it was up to 0.3 cases/100,000 persons/year in HBsAg+ individuals. The PAF and extra cases of HCC [31] were estimated by using the following formula:

$$\begin{array}{c} P_{cancer}=0.3\times P_{HBV+}+0.01\times \left(1-P_{HBV+}\right)\; \end{array}\;R_{AFTs}=P_{cancer}\times EDI\;\;\mathrm{PAF}=R_{AFTs}/R_{HCC\;}\;\mathrm{Case}=R_{AFTs}\times Population\;$$

where *P_cancer_* represents the average potency of HCC due to AFTs in the two populations (with and without HBV infection); *P_HBV+_* represents the prevalence rate of hepatitis-B-virus surface antigen (HBsAg+) in different provinces in China; *R_AFTs_* represents the HCC risk due to foodborne intake of AFTs; *EDI* represents total daily foodborne aflatoxin intake; *R_HCC_* represents the total incidence of HCC by all causes; PAF represents population attributable fraction; Case represents the extra-cases of HCC due to exposure to AFTs. The demographic data were obtained from China’s seventh census in 2020.

### 2.6. Estimation of Disability-Adjusted Life Years Attributed to Foodborne AFT Intake

Disability-adjusted life years (DALYs) is a metric to evaluate the burden of a particular disease. The direct approach was used to calculate the DALYs attributed to the extra-cases of HCC related to exposure to AFTs. Firstly, the average DALY number of each liver cancer case was calculated [26]. According to the China Cancer Registry Annual Report of 2019, the DisMod II software and template for calculating the burden of disease were used to calculate the DALYs of each HCC case. We calculated that the DALY number of each HCC case was 12.37 (detailed information is shown in Supplementary Figure S3). The total DALYs of HCC of aflatoxin-related origin was equal to the number of cases multiplied by 12.37. The DALY rate (per 100,000) of HCC attributed to foodborne intake was calculated in the last stage.

### 2.7. Sensitivity Analysis

Based on literature retrieval, we obtained the sample-size-weighted mean of AFTs in each region to calculate the national total levels of AFTs in foods. However, regions with a large sample size may skew the pooled estimates. We recalculated the AFTs’ concentration after excluding the region with the largest sample size of 4797. The results demonstrated that the recalculated pooled AFT concentration was similar to that before excluding the largest sample study (14.37 vs. 14.47 μg/kg).

### 2.8. Statistical Analysis

Uncertainty intervals were performed with Monte Carlo simulation using @RISK 7.6 (Palisade Corporation, Ithaca, NY, USA) to assess the variability of risk estimates. The appropriate probability distributions were used to describe the parameters. Monte Carlo simulations were performed considering 100,000 iterations to obtain probability distributions. We obtained stable outputs by enabling convergence testing in every 200 iterations. The 95% uncertainty intervals (UIs) were derived from the output values of the 2.5th and 97.5th percentiles.

DisMod II software and an Excel template recommended by the WHO for disease burden calculation were used to estimate DALYs [32]. ArcGIS software was employed for visualization. All statistical analyses were conducted using SPSS 20.0 (IBM Corp., Armonk, NY, USA). All tests were two-sided with a significance level of 0.05.

## 3. Results

### 3.1. AFT Concentration in Peanuts, Peanut Oil, Corn, and Corn Products in China

A total of 47 studies were collected with 16,604 unique pieces of AFT data from 31 provinces, autonomous regions, and municipalities of mainland China. Table 1 presents an overview of the occurrence data obtained for peanuts, peanut oil, corn, and corn products. The detailed data in various periods are shown in Supplementary Table S2. A total of 5800 (34.93%) samples were positive for aflatoxins among the whole samples, and the positive rate of AFTs in peanut oil was the highest (49.14%), followed by that in corn (29.10%). The positive rate of AFTs in peanut, peanut oil, and corn was positively correlated with the number of detected cases (*p* < 0.05), while there was non-correlation in the case of corn products (*p* > 0.05) (Supplementary Figure S4). The weighted mean AFT contamination in peanut, peanut oil, corn, and corn products was 8.07, 14.47, 25.76, and 1.19 μg/kg, respectively. In peanuts, Shaanxi province had the highest levels of contamination, with a mean AFT contamination of 34.50 μg/kg, followed by Hebei (21.42 μg/kg). Guangxi had the highest mean AFT contamination of peanut oil (31.92 μg/kg) and corn (148.57 μg/kg). The mean AFT contamination in corn was much higher than that in peanuts, peanut oil, and corn products. The contamination in corn products was relatively low, and the highest concentration was 8.06 μg/kg in Jiangsu. AFT contamination in Hebei, Shaanxi, Fujian, Guangxi, Henan, Hunan, and Yunnan exceeded the Codex Alimentarius Commission (CAC) limit standard (15.0 μg/kg) and the China National Food Safety Standard limit (20.0 μg/kg). The AFT contamination in corn in Guangxi exceeded the CAC limit standard by 8.8 times.

### 3.2. Consumption Data of Peanuts, Peanut Oil, Corn, and Corn Products in China

The daily consumption data of peanuts, peanut oil, corn, and corn products are shown in Table 2. The daily consumption of peanuts ranged from 0.23 to 5.34 g/day in the general population, with Liaoning (5.34 g/day) being the highest, followed by Shandong (4.67 g/day). The consumption level for peanut oil in Shandong was the highest (37.81 g/day), while levels in Anhui and Jiangsu were the lowest (0.02 g/day). The corn consumption level in Jilin (20.96 g/day) was the highest, and the corn product consumption level in Shaanxi (18.87 g/day) was the highest. The peanut and peanut oil consumption levels were higher in South China and coastal areas, while the corn and corn product consumption levels were higher in Northeast and North China.

### 3.3. Estimated Daily Intake of AFTs from Peanuts, Peanut Oil, Corn, and Corn Products in China

As is shown in Table 3, the estimated daily intake (*EDI*) of AFTs was calculated based on consumption data and contamination data. The mean *EDI* was estimated to range from 0.00 to 1.102, 0.00 to 5.251, and 0.00 to 18.447 ng/kg bw/day for peanuts, peanut oil, corn and corn products, respectively, and the national average *EDI* was estimated to be 4.018 ng/kg bw/day (95% UI: 0.721, 10.955). The AFT exposure from corn was the highest, being 26 times that of peanuts, 7.7 times that of peanuts, and 1.6 times that of peanut oil. For AFT exposure from corn, Guangxi province was the highest (18.447 ng/kg bw/day; 95% UI: 0.468, 68.055), followed by Henan province (2.383 ng/kg bw/day; 95% UI: 0.060, 8.486). For AFT exposure from peanut oil, Guangdong province was the highest (5.251 ng/kg bw/day; 95% UI: 0.133, 19.371), followed by Shandong province (4.673 ng/kg bw/day; 95% UI: 0.119, 17.239).

**Figure 1:**
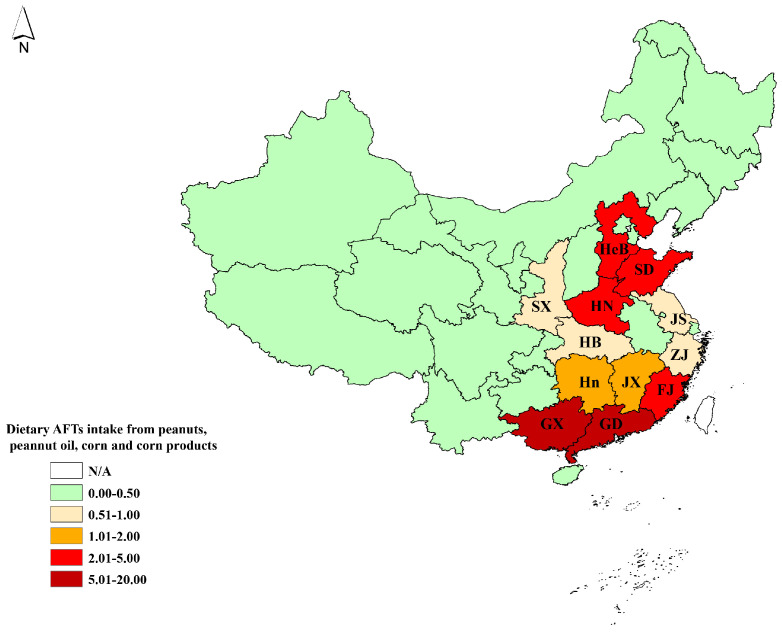
Distribution of the mean daily exposure to AFTs from peanuts, peanut oil, corn, and corn products. HeB: Hebei, HB: Hubei, SD: Shandong, SX: Shaanxi, HN: Henan; JS: Jiangsu; Hn: Hunan, JX: Jiangxi; ZJ: Zhejiang, FJ: Fujian, GD: Guangdong, and GX: Guangxi.

As is shown in Figure 1, the daily exposure to AFTs from peanuts, peanut oil, corn, and corn products was classified into five grades as 0.00–0.50, 0.51–1.00, 1.01–2.00, 2.01–5.00, and 5.01–20.00 ng/kg bw/day. Compared with other regions, South and East China residents had higher exposure to AFTs. Guangxi province was the highest, with up to 19.386 ng/kg bw/day, followed by Guangdong (5.812 ng/kg bw/day), Fujian (4.883 ng/kg bw/day), and Shandong (4.839 ng/kg bw/day). Southwest, Northwest, and Northeast China had exposures below 1.00 ng/kg bw/day.

### 3.4. Risk Characterization of Dietary Intake of AFTs

The HCC risk based on AFT intake was estimated according to the quantitative liver cancer risk approach proposed by JECFA. The HBsAg+ prevalence rate was derived from the China National Sero-epidemiological Survey of 2006 [33]. The estimated extra cases of HCC due to the exposure to AFTs in two groups are shown in Table 4. The overall average HCC risk caused by AFTs in the general Chinese population was 0.125 (95% UI: 0.022, 0.338) cases/100,000 persons/year. Guangxi province presented with the highest HCC risk of 0.959 (95% UI: 0.063, 3.423) cases/100,000 persons/year, followed by Guangdong province with 0.359 (95% UI: 0.028, 1.230) cases/100,000 persons/year. As published in the 2019 Annual Report of the China Cancer Registry, the incidence of liver cancer in China was 18.09 cases/100,000 persons/year in 2016, ranging from 15.98 to 21.41 cases/100,000 persons/year. The PAF of AFT exposure from peanuts, peanut oil, corn, and corn products accounted for 0.69% (95% UI: 0.12, 1.87) of the overall annual liver cancer incidence and ranged from 0.00% to 4.60% in different areas. Guangxi and Guangdong were the two highest, with contribution rates of 4.48% (95% UI: 0.30, 15.99) and 2.25% (95% UI: 0.17, 7.69), respectively (Table 4).

The MOE value was 99.6 of AFT exposure from peanuts, peanut oil, corn, and corn products for the general population in China. According to the estimate, 20 provinces/cities had MOE values below 10,000, with Guangxi province having the lowest, far less than the safe threshold (Table 4).

### 3.5. Estimated Disability-Adjusted Life Years Attributed to Dietary Exposure to AFTs in China

As shown in Table 5, the number of DALYs attributed to foodborne AFT intake was 21,625.08 (95% UI: 3878.31–58,968.04) in the Chinese population. When classified by region, the top three regions were Guangxi, Guangdong, and Shandong (5948.01, 5595.31, 1927. 92, respectively) (Supplementary Figure S5). The health loss per 100,000 persons due to AFT exposure ranged from 0.00 to 11.87 DALYs. The DALY rate was the highest in Guangxi province, with 11.87 (95% UI: 0.78, 42.34) per 100,000 persons, followed by Guangdong province (4.44 per 100,000 persons), and those with the lowest estimates were Neimongol, Guizhou, Xizang, Ningxia, Qinghai, and Xinjiang, with below 0.01 per 100,000 persons.

## 4. Discussion

Peanuts, corn, and their products were the primary sources of AFT exposures to people from foods. Our study found that these four foods had different levels of aflatoxin contamination. The detection rate for AFTs was the highest in peanut oil, while the concentration of AFTs was the highest in corn. A similar finding was also found by Ji et al. [34] that the positive rate was higher in peanut oil than in peanuts, as aflatoxin detected in peanut oil often comes from contaminated peanuts. The weighted mean of the concentration of AFTs in corn was higher than that of the other three foods, i.e., 25.76 μg/kg, exceeding the ML of 20 μg/kg in China. Gao et al. [35] reported that the mean concentration of AFTs was 44.04 μg/kg, and the positive rate was 75.63% of 279 corn samples from six provinces in China. Similar to the case in Indonesia, the overall level of aflatoxins in corn was higher than that in peanuts [36]. It may be that corn, once infected with *Aspergillus flavus*, is more likely to produce aflatoxin. Since the carcinogenic effect of aflatoxin is not governed by a threshold, attention should be paid to contamination with AFTs, e.g., either peanut oil with a high positive rate or corn with a high aflatoxin concentration. Furthermore, the positive rate of AFTs positively correlated with the number of detected cases for peanut, peanut oil, and corn, indicating that AFT contamination may be more severe than detected.

Although AFTs occur naturally, factors such as climate, inappropriate storage, and food processing contribute to AFT contamination in foods. Firstly, climatic factors are important for AFT contamination levels. Ding et al. [37] reported that, as the latitude goes up, the higher temperature and relative humidity might increase the risk of peanut contamination by *Aspergillus* and aflatoxins. Battilani et al. [38] reported that the concentration of aflatoxin B1 increased significantly in maize in Europe when the temperature increased by 2 °C in their prediction model. According to the review of Assuncao et al. [39], the potential consequence of climate change on the health effects of aflatoxins exposure should be monitored in Portugal and Europe. In the present study, the regions with subtropical climates, such as Guangdong, Guangxi, and Fujian, were found to be more conducive to the growth of aflatoxins due to their high temperature and humid environment. Secondly, rural households usually store post-harvest peanuts and corn in open bags on the ground, leading to fungal infection [40]. Unpackaged food from small workshops or farmer’s markets is also easily contaminated [40,41]. Thirdly, homemade food is widely consumed in many rural areas in China, e.g., peanut oil; the simple traditional procedures cannot degrade AFTs, and the poor-quality machines may lead to AFT contamination during the production process [42]. In addition, genetic polymorphisms of toxigenic strains may affect aflatoxin production [43].

Risk assessment results demonstrate that the attributable risk of liver cancer caused by AFTs was relatively low in China, while some areas still had high health risks. The PAF of AFT-induced liver cancer was 0.69% in our study, far lower than that in Liu and Wu’s study [15]. They reported that aflatoxin played a causative role in 4.6–28.2% of all global HCC cases in 2010 [15]. In Qin’s study, the liver cancer risk due to peanuts and peanut oil consumption contributed 0.30–0.33% to the annual liver cancer incidence in China [14]. Our estimated PAF was higher than that in Qin’s study because only peanuts and peanut oil were included in their research. Hepatitis B vaccine and staple-food replacement could account for the low risk of AFTs associated with HCC in China now. Hepatitis B vaccination increased to 60% coverage, and the HBsAg prevalence decreased by up to 12% annually from 2000 onward [19]. Considering the combined effect of hepatitis B infection and aflatoxin on HCC, reducing the incidence of hepatitis B could reduce the risk of AFT-induced HCC. Replacing maize with rice in the areas with high AFT contamination was considered one of the successful measures to reduce liver cancer in areas in China where the aflatoxin pollution was severe in corn [44]. However, the PAF was 2.25% and 4.48% in Guangdong and Guangxi provinces, respectively, which revealed a relatively higher risk of AFT exposure. Furthermore, the MOE values in 20 regions were below 10,000 and should be a cause for concern.

The estimated number of DALYs in our present study was lower than the reported estimates. We estimated that the DALY number and DALY rate related to AFT exposure were 21,625.08 and 1.53 per 100,000 persons, respectively. The Foodborne Disease Burden Epidemiology Reference Group (FERG) estimated that the median DALY was 17 per 100,000 persons caused by aflatoxins in WPR B (including China) in 2010 [45]. Although the estimated burden of aflatoxins was not very high in the present study, more than 20,000 years of life could be saved if some precise preventive measures could reduce the exposure to AFTs in the Chinese population. Hence, control measures are necessary to minimize aflatoxin exposure, thereby saving years of life lost in relation to AFT contamination.

Although the present study has provided a national-level estimation of AFT exposure and risk, there are several limitations. Firstly, the consumption data were obtained from the China National Nutrition and Health Survey (CNNHS) of 2002. The dietary pattern might have changed due to socioeconomic development during the past decade. Consequently, using the previous consumption data would result in under- or overestimation of the dietary AFT exposure [8]. Secondly, the typical heterogeneous distribution of aflatoxins may lead to inaccurate exposure estimations [8]. Furthermore, while other food categories, including rice, nuts, or beans, might also be contaminated with AFTs, the disease burden of this AFT exposure cannot be assessed due to the insufficient data. A more comprehensive estimation for the overall dietary AFT exposure of the Chinese population needs to be done in future research. Finally, although our design was accurate and reasonable, some better approaches that systematically and comprehensively collecte representative data for synthesis and analysis could be implemented to conduct this type of study in the future.

## 5. Conclusions

In conclusion, our study indicated that the disease burden of dietary AFT intake from peanuts, peanut oil, corn, and corn products was low in the Chinese population. Nevertheless, some subpopulations still have high AFT-related health risks in several areas. Continuous efforts and targeted strategies are needed to control AFT contamination, thereby reducing the disease burden of AFTs.

## Figures and Tables

**Table 1:** AFT concentration in peanuts, peanut oil, corn, and corn products in different areas of China from 2010 to 2020.

Province	Peanut			Peanut Oil			Corn			Corn Products		
	N	Positive N (%)	Means * (μg/kg)	N	Positive N (%)	Means * (μg/kg)	N	Positive N (%)	Means * (μg/kg)	N	Positive N (%)	Means * (μg/kg)
Total	5092	1353 (26.57)	8.07	7290	3583 (49.15)	14.47	1746	508 (29.10)	25.76	2476	356 (14.38)	1.19
North												
Beijing	15	3 (20.00)	0.71									
Hebei	40	18 (45.00)	21.42	82	35 (42.68)	7.08	92	78 (84.78)	8.96			
Neimongol	15	0 (0.00)	0.34									
Shanxi	16	0 (0.00)	4.00				13	8 (61.54)	1.83			
Tianjin	219	46 (21.00)	7.47				200	67 (33.50)	8.98			
Northeast												
Heilongjiang	10	2 (20.00)	0.41				18	10 (55.56)	0.57			
Jilin	25	3 (12.00)	1.28	13	0 (0.00)	0.5						
Liaoning	130	13 (10.00)	0.34	50	2 (4.00)	1.68						
East												
Anhui	706	232 (32.86)	9.25				30	15 (50.00)	1.06			
Fujian	284	31 (10.92)	14.02	166	105 (63.25)	27.42	16	6 (37.50)	0.57			
Jiangsu	421	124 (29.45)	3.33	33	8 (24.24)	1.31				106	49 (46.23)	8.06
Jiangxi	482	168 (34.85)	10.81	38	12 (31.58)	10.29	11	5 (45.45)	0.43			
Shandong	201	18 (8.96)	1.95	326	103 (31.60)	6.78	689	159 (23.08)	6.17	371	125 (33.69)	1.44
Shanghai	97	23 (23.71)	12.09				19	7 (36.84)	1.26			
Zhejiang	50	9 (18.00)	9.90	363	31 (8.54)	0.26				125	1 (0.80)	0.07
Central												
Henan	107	44 (41.12)	5.89	270	43 (15.93)	1.99	40	32 (80.00)	50.32	659	61 (9.26)	0.81
Hubei	424	171 (40.33)	6.55	67	2 (2.99)	1.60	20	12 (60.00)	3.4			
Hunan	468	160 (34.19)	13.49				10	7 (70.00)	22.1			
South												
Guangdong	302	85 (28.15)	11.73	4797	2623 (54.68)	14.42				548	48 (8.76)	0.26
Guangxi	56	26 (46.43)	7.78	844	492 (58.29)	31.92	230	39 (16.96)	148.57	58	30 (51.72)	3.03
Hainan	46	5 (10.87)	0.47				3	2 (66.67)	6.15			
Southwest												
Chongqing	136	55 (40.44)	0.97	73	28 (38.36)	0.63	16	16 (100.00)	6.44	63	24 (38.10)	0.95
Guizhou	23	1 (4.35)	0.21	17	11 (64.71)	1.02						
Sichuan	535	77 (14.39)	5.75									
Xizang	23	0 (0.00)	0.05									
Yunnan	70	11 (15.71)	0.56	28	6 (21.43)	3.44	14	11 (78.57)	65.74	292	>6 (>2.05)	1.83
Northwest												
Gansu	63	6 (9.52)	0.59				17	3 (17.65)	0.58			
Ningxia	10	5 (50.00)	0.89									
Qinghai	14	0 (0.00)	1.20									
Shaanxi	64	17 (26.56)	34.5	123	82 (66.67)	1.14	308	31 (10.06)	1.54	254	18 (7.09)	0.37
Xinjiang	40	0 (0.00)	0.11									

* Weighted means; N: number of samples.

**Table 2:** The mean consumption levels of peanuts, peanut oil, corn, and corn products in different areas of China (g/day).

Province	Consumption Levels of Peanuts (g/Day)	Consumption Levels of Peanut Oil (g/Day)	Consumption Levels of Corn (g/Day)	Consumption Levels of Corn Products (g/Day)
Total	2.19	5.49	4.98	4.17
North				
Beijing	3.00	26.10	9.56	10.17
Hebei	3.24	15.05	2.41	14.36
Neimongol	0.88	0.70	9.25	1.42
Shanxi	1.00	0.81		
Tianjin	4.24	7.34		
Northeast				
Heilongjiang	1.47		7.11	2.45
Jilin	1.73		20.96	1.53
Liaoning	5.34	0.26	5.95	6.06
East				
Anhui	1.56	0.02		
Fujian	3.43	7.25		
Jiangsu	4.27	0.02	1.69	4.80
Jiangxi	1.90	4.35	1.35	0.01
Shandong	4.67	37.81		
Shanghai	2.51	0.46		
Zhejiang	3.78	0.18	6.19	0.17
Central				
Henan	2.22	7.04	2.93	7.51
Hubei	3.02	5.28	1.82	2.25
Hunan	1.99	0.39	2.71	0.10
South				
Guangdong	2.55	19.43		
Guangxi	3.00	0.69	6.62	1.23
Hainan	1.83	10.24		
Southwest				
Chongqing	1.25	0.05		
Guizhou	0.68	0.63	0.42	0.39
Sichuan	1.38	0.22	1.35	0.52
Xizang	0.23	0.59		
Yunnan	3.50	0.50		
Northwest				
Gansu	0.42		5.43	3.14
Ningxia	0.49	2.43	3.40	0.02
Qinghai	0.36			
Shaanxi	1.00	0.30	0.42	18.87
Xinjiang	0.94	0.09		

**Table 3:** AFT intake through peanuts, peanut oil, corn, and corn products in China (ng/kg bw/day).

Province	AFT Exposure from Peanuts (95% UI)	AFT Exposure from Peanut Oil (95% UI)	AFT Exposure from Corn (95% UI)	AFT Exposure from Corn Products (95% UI)	AFT Exposure from Peanut, Peanut Oil, Corn, and Corn Products (95% UI)
Total	0.308 (0.008, 1.138)	1.385 (0.035, 5.113)	2.238 (0.057, 8.333)	0.086 (0.002, 0.322)	4.018 (0.721, 10.955)
North					
Beijing	0.033 (0.001, 0.032)				0.033 (0.001, 0.032)
Hebei	1.102 (0.027, 2.993)	1.691 (0.043, 6.239)	0.343 (0.009, 1.251)		3.136 (0.548, 7.861)
Neimongol	0.005 (0.000, 0.012)				0.005 (0.000, 0.012)
Shanxi	0.076 (0.001, 0.073)				0.076 (0.001, 0.073)
Tianjin	0.499 (0.013, 1.842)				0.499 (0.013, 1.842)
Northeast					
Heilongjiang	0.010 (0.000, 0.035)		0.065 (0.001, 0.111)		0.075 (0.006, 0.123)
Jilin	0.035 (0.001, 0.050)	0.000 (0.000, 0.000)			0.035 (0.001, 0.050)
Liaoning	0.029 (0.001, 0.106)	0.007 (0.000, 0.016)			0.036 (0.003, 0.113)
East					
Anhui	0.27 (0.005, 0.399)				0.270 (0.005, 0.399)
Fujian	0.951 (0.024, 3.51)	3.932 (0.100, 14.502)			4.883 (0.496, 15.664)
Jiangsu	0.230 (0.006, 0.847)	0.000 (0.000, 0.001)		0.625 (0.016, 2.305)	0.855 (0.094, 2.606)
Jiangxi	0.367 (0.009, 1.355)	0.801 (0.019, 2.195)	0.010 (0.000, 0.019)		1.178 (0.135, 2.688)
Shandong	0.166 (0.004, 0.595)	4.673 (0.119, 17.239)			4.839 (0.250, 17.307)
Shanghai	0.477 (0.012, 1.761)				0.477 (0.012, 1.761)
Zhejiang	0.633 (0.016, 2.336)	0.001 (0.000, 0.003)			0.634 (0.017, 2.337)
Central					
Henan	0.211 (0.005, 0.515)	0.227 (0.006, 0.835)	2.383 (0.060, 8.486)	0.098 (0.002, 0.363)	2.919 (0.394, 8.968)
Hubei	0.337 (0.0085, 1.243)	0.144 (0.003, 0.171)	0.105 (0.003, 0.260)		0.586 (0.094, 1.399)
Hunan	0.487 (0.012, 1.796)		1.086 (0.026, 2.875)		1.572 (0.179, 3.543)
South					
Guangdong	0.561 (0.014, 2.068)	5.251 (0.133, 19.371)			5.812 (0.450, 19.904)
Guangxi	0.438 (0.011, 1.615)	0.413 (0.010, 1.524)	18.447 (0.468, 68.055)	0.070 (0.002, 0.258)	19.368 (1.275, 69.105)
Hainan	0.016 (0.000, 0.058)				0.016 (0.000, 0.058)
Southwest					
Chongqing	0.024 (0.001, 0.089)	0.001 (0.000, 0.001)			0.025 (0.001, 0.089)
Guizhou	0.003 (0.000, 0.007)	0.012 (0.000, 0.034)			0.015 (0.001, 0.036)
Sichuan	0.138 (0.003, 0.431)				0.138 (0.003, 0.431)
Xizang	0.000 (0.000, 0.000)				0.000 (0.000, 0.000) 0.000 (0.000, 0.000)
Yunnan	0.038 (0.001, 0.111)	0.033 (0.001, 0.084)			0.071 (0.008, 0.148)
Northwest					
Gansu	0.004 (0.000, 0.015)		0.055 (0.001, 0.094)		0.06 (0.003, 0.099)
Ningxia	0.008 (0.000, 0.026)				0.008 (0.000, 0.026)
Qinghai	0.008 (0.000, 0.023)				0.008 (0.000, 0.023)
Shaanxi	0.579 (0.015, 2.133)	0.006 (0.000, 0.017)	0.011 (0.000, 0.04)	0.117 (0.003, 0.432)	0.713 (0.082, 2.304)
Xinjiang	0.002 (0.000, 0.002)				0.002 (0.000, 0.002)

AFTs include four aflatoxins: AFB1, AFB2, AFG1, and AFG2; UI: uncertainty interval.

**Table 4:** The HCC risk, PAF, and MOE of AFT exposure from peanuts, peanut oil, corn, and corn products.

Province	Estimated Annual HCC/100,000 (HBV+)	Estimated Annual HCC/100,000 (HBV−)	HCC Risk of AFT Exposure *, 95% UI	HCC Incidence #	PAF% ^, 95% UI	MOE
Total	0.022	0.009	0.125 (0.022, 0.338)	18.07	0.69 (0.12, 1.87)	99.6
North						
Beijing	0.014	0.010	0.001 (0.000, 0.001)	15.98	0.00 (0.00, 0.00)	12,121.2
Hebei	0.013	0.010	0.072 (0.013, 0.181)	15.98	0.45 (0.08, 1.13)	127.6
Neimongol	0.019	0.009	0.000 (0.000, 0.000)	21.41	0.00 (0.00, 0.00)	80,000.0
Shanxi	0.019	0.009	0.002 (0.000, 0.002)	19.12	0.01 (0.00, 0.01)	5263.2
Tianjin	0.017	0.009	0.013 (0.000, 0.049)	15.98	0.08 (0.00, 0.31)	801.6
Northeast						
Heilongjiang	0.032	0.009	0.003 (0.000, 0.005)	19.12	0.02 (0.00, 0.03)	5333.3
Jilin	0.026	0.009	0.001 (0.000, 0.002)	19.12	0.01 (0.00, 0.01)	11,428.6
Liaoning	0.039	0.009	0.002 (0.000, 0.005)	15.98	0.01 (0.00, 0.03)	11,111.1
East						
Anhui	0.023	0.009	0.009 (0.000, 0.013)	19.12	0.05 (0.00, 0.07)	1481.5
Fujian	0.051	0.008	0.291 (0.030, 0.933)	15.98	1.82 (0.18, 5.84)	81.9
Jiangsu	0.020	0.009	0.025 (0.003, 0.076)	15.98	0.16 (0.02, 0.47)	467.8
Jiangxi	0.046	0.008	0.065 (0.007, 0.148)	19.12	0.34 (0.04, 0.77)	339.6
Shandong	0.022	0.009	0.154 (0.008, 0.549)	15.98	0.96 (0.05, 3.44)	82.7
Shanghai	0.022	0.009	0.015 (0.000, 0.055)	15.98	0.09 (0.00, 0.35)	838.6
Zhejiang	0.033	0.009	0.026 (0.001, 0.097)	15.98	0.16 (0.00, 0.61)	630.9
Central						
Henan	0.036	0.009	0.130 (0.018, 0.399)	19.12	0.68 (0.09, 2.09)	137.0
Hubei	0.033	0.009	0.025 (0.004, 0.059)	19.12	0.13 (0.02, 0.31)	682.6
Hunan	0.023	0.009	0.051 (0.006, 0.114)	19.12	0.27 (0.03, 0.60)	254.5
South						
Guangdong	0.054	0.008	0.359 (0.028, 1.230)	15.98	2.25 (0.17, 7.69)	68.8
Guangxi	0.041	0.009	0.959 (0.063, 3.423)	21.41	4.48 (0.30, 15.99)	20.7
Hainan	0.053	0.008	0.001 (0.000, 0.004)	15.98	0.01 (0.00, 0.02)	25,000.0
Southwest						
Chongqing	0.034	0.009	0.001 (0.000, 0.004)	21.41	0.00 (0.00, 0.02)	16,000.0
Guizhou	0.017	0.009	0.000 (0.000, 0.001)	21.41	0.00 (0.00, 0.00)	26,666.7
Sichuan	0.031	0.009	0.006 (0.000, 0.017)	21.41	0.03 (0.00, 0.08)	2898.6
Xizang	0.052	0.008	0.000 (0.000, 0.000)	21.41	0.00 (0.00, 0.00)	N/A
Yunnan	0.020	0.009	0.002 (0.000, 0.004)	21.41	0.01 (0.00, 0.02)	5633.8
Northwest						
Gansu	0.024	0.009	0.002 (0.000, 0.003)	21.41	0.01 (0.00, 0.02)	6666.7
Ningxia	0.037	0.009	0.000 (0.000, 0.001)	21.41	0.00 (0.00, 0.01)	50,000.0
Qinghai	0.025	0.009	0.000 (0.000, 0.001)	21.41	0.00 (0.00, 0.00)	50,000.0
Shaanxi	0.027	0.009	0.026 (0.003, 0.084)	21.41	0.12 (0.01, 0.39)	561.0
Xinjiang	0.021	0.009	0.000 (0.000, 0.000)	21.41	0.00 (0.00, 0.00)	200,000.0

HCC: hepatocellular carcinoma; HBV+: hepatitis B surface antigen positive; HBV−: hepatitis B surface antigen negative; MOE: margin of exposure; AFTs include four aflatoxins: AFB1, AFB2, AFG1, and AFG2; UI: uncertainty interval. * AFT exposure from peanuts, peanut oil, corn, and corn products (cases/100,000 persons/year); # liver cancer incidence in 2016 (cases/100,000 persons/year); ^ population attributable fraction.

**Table 5:** Estimated DALY of HCC due to AFTs in China in 2020.

Province	Population (Million)	Annual HCC Cases (HBV+), 95% UI	Annual HCC Cases (HBV−), 95% UI	DALY Number, 95% UI	DALY Rate (per 100,000), 95% UI
Total	1,411,778,724	1221.72 (219.11, 3331.44)	526.46 (94.42, 1435.58)	21,625.08 (3878.31, 58,968.04)	1.53 (0.27, 4.18)
North					
Beijing	21,893,095	0.10 (0.00, 0.10)	0.07 (0.00, 0.07)	2.10 (0.03, 2.06)	0.01 (0.00, 0.01)
Hebei	74,610,235	31.52 (5.50, 79.01)	22.35 (3.90, 56.02)	666.27 (116.29, 1670.19)	0.89 (0.16, 2.24)
Neimongol	24,049,155	0.02 (0.00, 0.06)	0.01 (0.00, 0.03)	0.41 (0.01, 1.06)	0.00 (0.00, 0.00)
Shanxi	34,915,616	0.51 (0.01, 0.49)	0.25 (0.00, 0.24)	9.41 (0.15, 9.02)	0.03 (0.00, 0.03)
Tianjin	13,866,009	1.18 (0.03, 4.35)	0.65 (0.02, 2.41)	22.66 (0.58, 83.62)	0.16 (0.00, 0.60)
Northeast					
Heilongjiang	31,850,088	0.76 (0.06, 1.25)	0.21 (0.02, 0.35)	12.01 (0.94, 19.75)	0.04 (0.00, 0.06)
Jilin	24,073,453	0.22 (0.00, 0.31)	0.08 (0.00, 0.11)	3.64 (0.07, 5.19)	0.02 (0.00, 0.02)
Liaoning	42,591,407	0.59 (0.06, 1.87)	0.13 (0.01, 0.42)	9.00 (0.83, 28.34)	0.02 (0.00, 0.07)
East					
Anhui	61,027,171	3.83 (0.08, 5.66)	1.52 (0.03, 2.25)	66.24 (1.32, 97.78)	0.11 (0.00, 0.16)
Fujian	41,540,086	103.99 (10.56, 333.59)	16.82 (1.71, 53.95)	1494.44 (151.8, 4793.78)	3.60 (0.37, 11.54)
Jiangsu	84,748,016	14.32 (1.58, 43.66)	6.76 (0.75, 20.63)	260.78 (28.79, 795.19)	0.31 (0.03, 0.94)
Jiangxi	45,188,635	24.73 (2.81, 56.41)	4.50 (0.51, 10.27)	361.54 (41.15, 824.73)	0.80 (0.09, 1.83)
Shandong	101,527,453	110.40 (5.705, 394.83)	45.45 (2.35, 162.56)	1927.92 (99.61, 6894.8)	1.90 (0.10, 6.79)
Shanghai	24,870,895	2.63 (0.07, 9.72)	1.10 (0.03, 4.06)	46.15 (1.17, 170.31)	0.19 (0.00, 0.69)
Zhejiang	64,567,588	13.37 (0.355, 49.25)	3.65 (0.10, 13.45)	210.48 (5.61, 775.52)	0.33 (0.01, 1.20)
Central					
Henan	99,365,519	103.56 (13.97, 318.1)	25.56 (3.45, 78.5)	1597.17 (215.46, 4905.94)	1.61 (0.22, 4.94)
Hubei	57,752,557	11.25 (1.81, 26.82)	3.01 (0.48, 7.19)	176.4 (28.31, 420.64)	0.31 (0.05, 0.73)
Hunan	66,444,864	24.04 (2.74, 54.17)	9.65 (1.10, 21.74)	416.67 (47.48, 938.8)	0.63 (0.07, 1.41)
South					
Guangdong	126,012,510	392.17 (30.34, 1343.11)	60.16 (4.66, 206.05)	5595.31 (432.94, 19,162.95)	4.44 (0.34, 15.21)
Guangxi	50,126,804	396.99 (26.15, 1416.44)	83.85 (5.52, 299.19)	5948.01 (391.73, 21,222.35)	11.87 (0.78, 42.34)
Hainan	10,081,232	0.08 (0.00, 0.31)	0.01 (0.00, 0.05)	1.21 (0.03, 4.45)	0.01 (0.00, 0.04)
Southwest					
Chongqing	32,054,159	0.27 (0.01, 0.96)	0.07 (0.00, 0.25)	4.15 (0.17, 15.01)	0.01 (0.00, 0.05)
Guizhou	38,562,148	0.10 (0.01, 0.24)	0.05 (0.00, 0.13)	1.86 (0.16, 4.54)	0.00 (0.00, 0.01)
Sichuan	83,674,866	3.63 (0.09, 11.34)	1.03 (0.03, 3.23)	57.67 (1.44, 180.11)	0.07 (0.00, 0.22)
Xizang	3,648,100	0.00 (0.00, 0.00)	0.00 (0.00, 0.00)		0.00 (0.00, 0.00)
Yunnan	47,209,277	0.68 (0.07, 1.42)	0.31 (0.03, 0.65)	12.24 (1.32, 25.59)	0.03 (0.00, 0.05)
Northwest					
Gansu	25,019,831	0.35 (0.02, 0.59)	0.14 (0.01, 0.23)	6.08 (0.35, 10.07)	0.02 (0.00, 0.04)
Ningxia	7,202,654	0.02 (0.00, 0.07)	0.00 (0.00, 0.02)	0.31 (0.01, 1.07)	0.00 (0.00, 0.01)
Qinghai	5,923,957	0.01 (0.00, 0.03)	0.00 (0.00, 0.01)	0.20 (0.00, 0.56)	0.00 (0.00, 0.01)
Shaanxi	39,528,999	7.67 (0.88, 24.81)	2.56 (0.29, 8.28)	126.58 (14.52, 409.32)	0.32 (0.04, 1.04)
Xinjiang	25,852,345	0.01 (0.00, 0.01)	0.00 (0.00, 0.01)	0.19 (0.00, 0.22)	0.00 (0.00, 0.00)

DALY: disability-adjusted life years; HCC: hepatocellular carcinoma; HBV+: hepatitis B surface antigen-positive; HBV−: hepatitis B surface antigen-negative; AFTs include four aflatoxins: AFB1, AFB2, AFG1, and AFG2; UI: uncertainty interval.

## Data Availability

Detailed data are available from the authors.

## Supplementary Materials

The following supporting information can be downloaded at: https://www.mdpi.com/article/10.3390/nu14051027/s1, Figure S1: The framework of study design; Figure S2: Flow chart of literature retrieval about aflatoxin contaminate; Figure S3: The main interface of the DALY calculator; Figure S4: Correlation between the AFTs positive rate and the total number of test samples; Figure S5: DALY of HCC associated with AFTs exposure from peanuts, peanut oil, corn, and corn products; Table S1: Retrieval strategies and results about aflatoxins in China in 2010–2020; Table S2: Relevant information of aflatoxin contaminate in peanuts; Table S3: Relevant information of aflatoxin contaminate in peanut oil; Table S4: Relevant information of aflatoxin contaminate in corn and corn product. (References [2,14,40,42,46,47,48,49,50,51,52,53,54,55,56,57,58,59,60,61,62,63,64,65,66,67,68,69,70,71,72,73,74,75,76,77,78,79,80,81,82,83,84,85,86,87,88] are cited in the supplementary materials).
