# Supplementary material for: Burden of Disease Associated with Dietary Exposure to Aflatoxins in China in 2020
Source: Nutrients. 2022 Feb 28;14(5):1027. doi: 10.3390/nu14051027 (PMC8912679; doi:10.3390/nu14051027)
Supplement: Supplementary file 1 [file nutrients-14-01027-s001.zip › nutrients-1567712-supplementary.pdf]

Supplemental Material

Table S1. Retrieval strategies and results about aflatoxins in China in 2010-2020.

Databases [#]	Retrieval Strategy	Number of Articles
Pubmed	aflatoxin AND (exposure OR "risk assessment" OR contaminants) AND China AND ("2010/01/01" : "2020/12/31")	696
CNKI (In Chinese)	(TKA= aflatoxin OR TKA=AFB1) AND (TKA= exposure OR TKA=risk OR TKA=food OR TKA= assessment OR TKA= contaminants) * Date: 2010-2020	2427
WAN FANG (In Chinese)	Title or Keyword: (aflatoxin OR AFB1) * Title or Keyword: (exposure OR risk OR food OR assessment OR contaminants) * Language: Chinese * Date: 2010-2020	2090
CBMdisc (In Chinese)	"(aflatoxin OR AFB1) AND (exposure OR risk OR food OR assessment OR contaminants)" * Date: 2010-2020"	426

[#] China National Knowledge Internet (CNKI), Wan Fang, and China Biology Medicine Disc (CBMdisc) are the professional academic databases of China. TKA: Title or Keyword or Abstract.

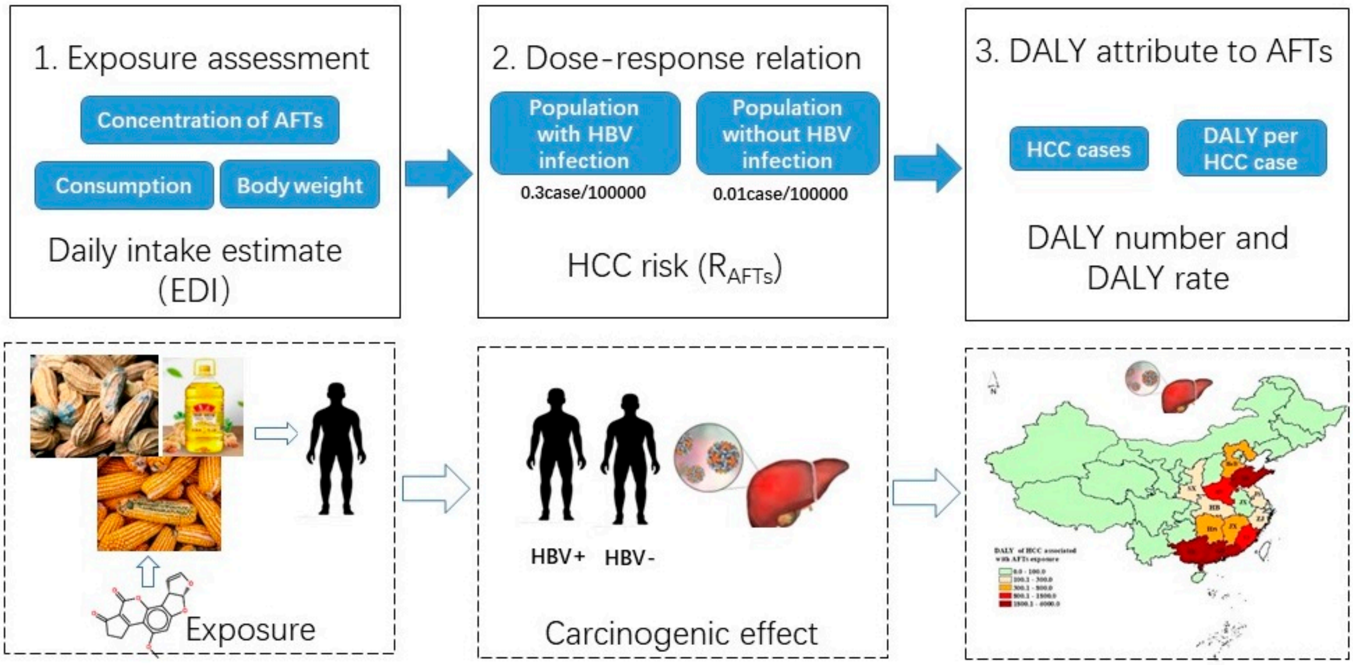

Figure S1. The framework of study design.

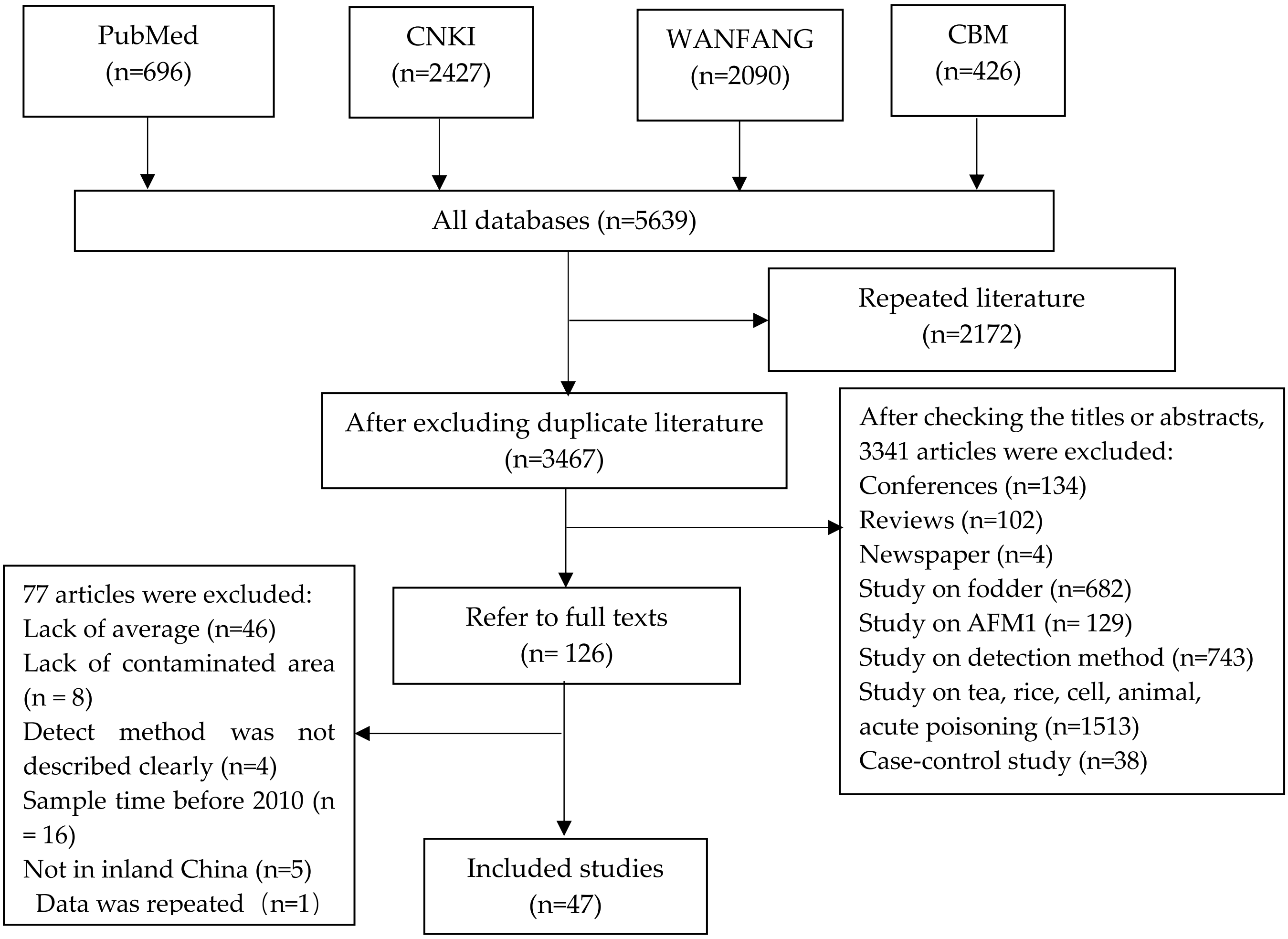

Figure S2. Flow chart of literature retrieval about aflatoxin contaminate. Notes: The retrieval results shown that we identified 696 titles from PubMed, 2427 titles from CNKI, 2090 titles from WANFANG, and 426 titles from CBMdisc. We filter titles and abstracts to identify articles that meet the requirements. There were 2172 repeated studies. After deduplication, 3467 articles were screened. Then checked the titles and abstracts, 3341 articles were excluded because some conferences, reviews, reports, newspapers, or the research content were about fodder, AFM1, detection method, and other contents that did not conform to this study. After a full-text review, we removed 77 papers, due to the lack of average, contaminated area data, the time of sample was not in conformity with our requirements. Finally, we included 47 studies that provided information about the contamination of AFTs in peanut, peanut oil, corn and corn product. We used a standardized database to extract the title, journal, location, study year, sample time, detection methods.

DALY calculator

DALY was calculated as the sum of the years of life lost (YLL) and the years lived with disability (YLD). The DALY was estimated by using the following formula:

DALYs =YLL +YLD

YLL=N * L

YLD= I * DW * L'

DALYs meant Disability-adjusted life years; YLL meant the years of life lost; YLD meant the years lived with disability; N meant number of deaths; L meant the remaining life expectancy at death; P meant prevalence; I meant the number of new cases; DW meant disability weights; L' meant the time of disease duration. The World Health Organization provided the templates to calculate DALY. But firstly, we need to calculate the L' .So we used Dismod II software to estimate the L'. Then we substituted population data, liver cancer incidence data, death data, duration and other parameters into the DALY calculation template to obtain the total DALY number. The average number of DALYs in each case was obtained by dividing the number of DALYs by the total number of cases.

Dismod II software was developed by WHO (download from http://www.epigear.com/index_files/dismod_ii.html), which was an epidemiological model and could be used to calculate the parameters related to DALY. **The main interface of the DALY calculator was shown below:**

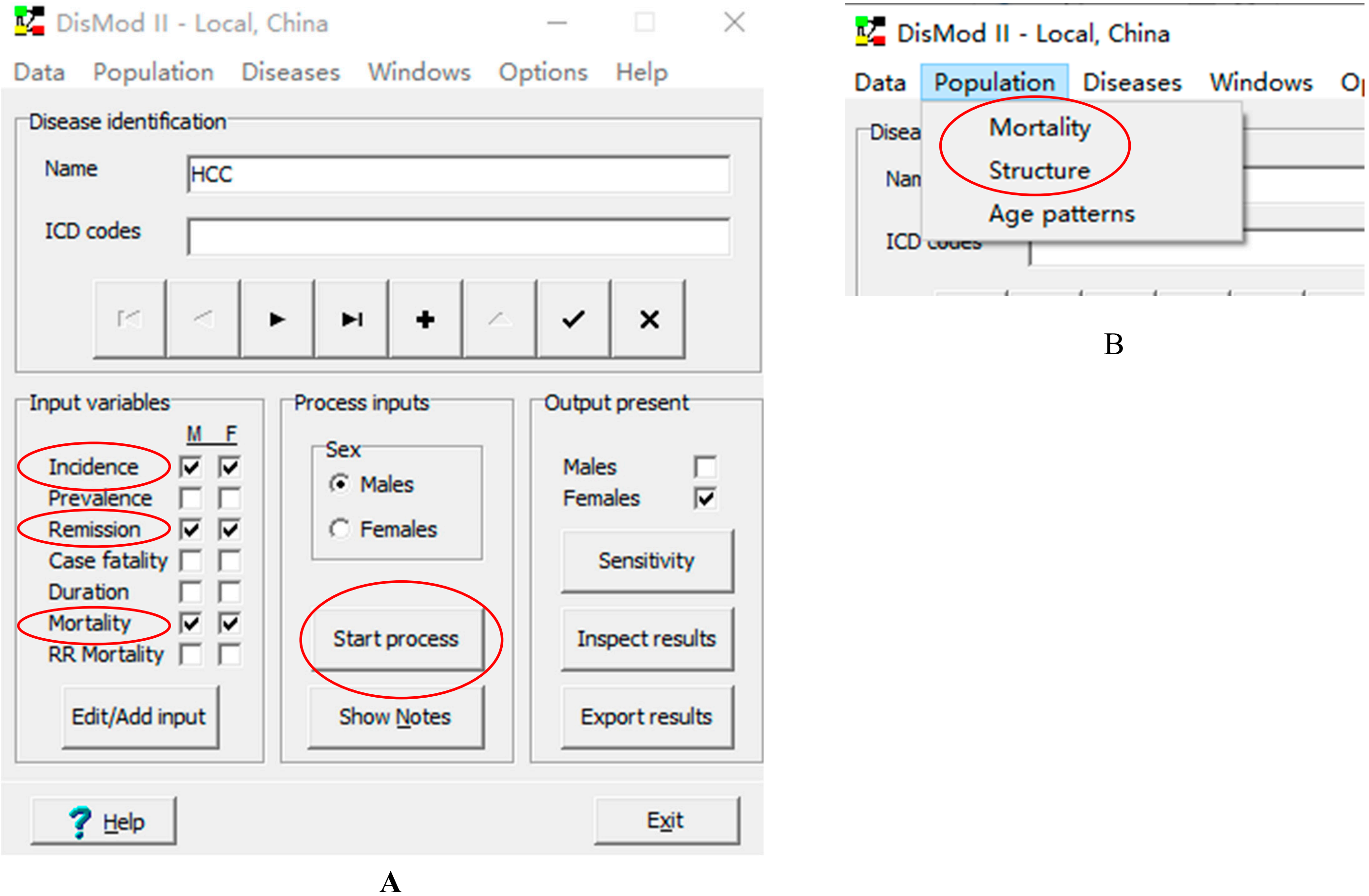

Figure S3. The main interface of the DALY calculator. (**A**) the main interface; (**B**) total population mortality and structure input interface input incidence， remission, mortality, and the total population mortality and structure, and then click on the “start process”.

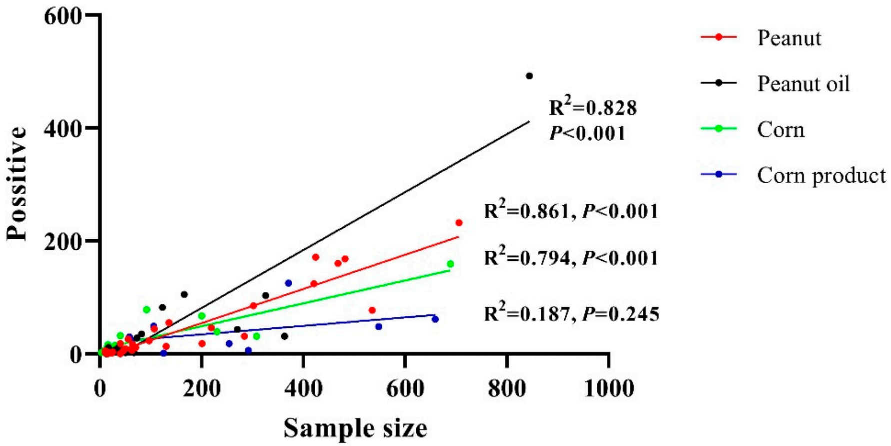

Peanut	Peanut oil	Corn	Corn product
Y = 0.3027*X - 6.073	Y = 0.5128*X - 21.23	Y = 0.2022*X + 8.606	Y = 0.07515*X + 19.55

Figure S4. Correlation between the AFTs positive rate and the total number of test samples. The positive number of aflatoxins in peanut, peanut oil, and corn were correlated with the number of tested samples (*P*<0.001). While the Positive number of aflatoxins in corn product was not associated with the number of tested samples (*P*=0.245).

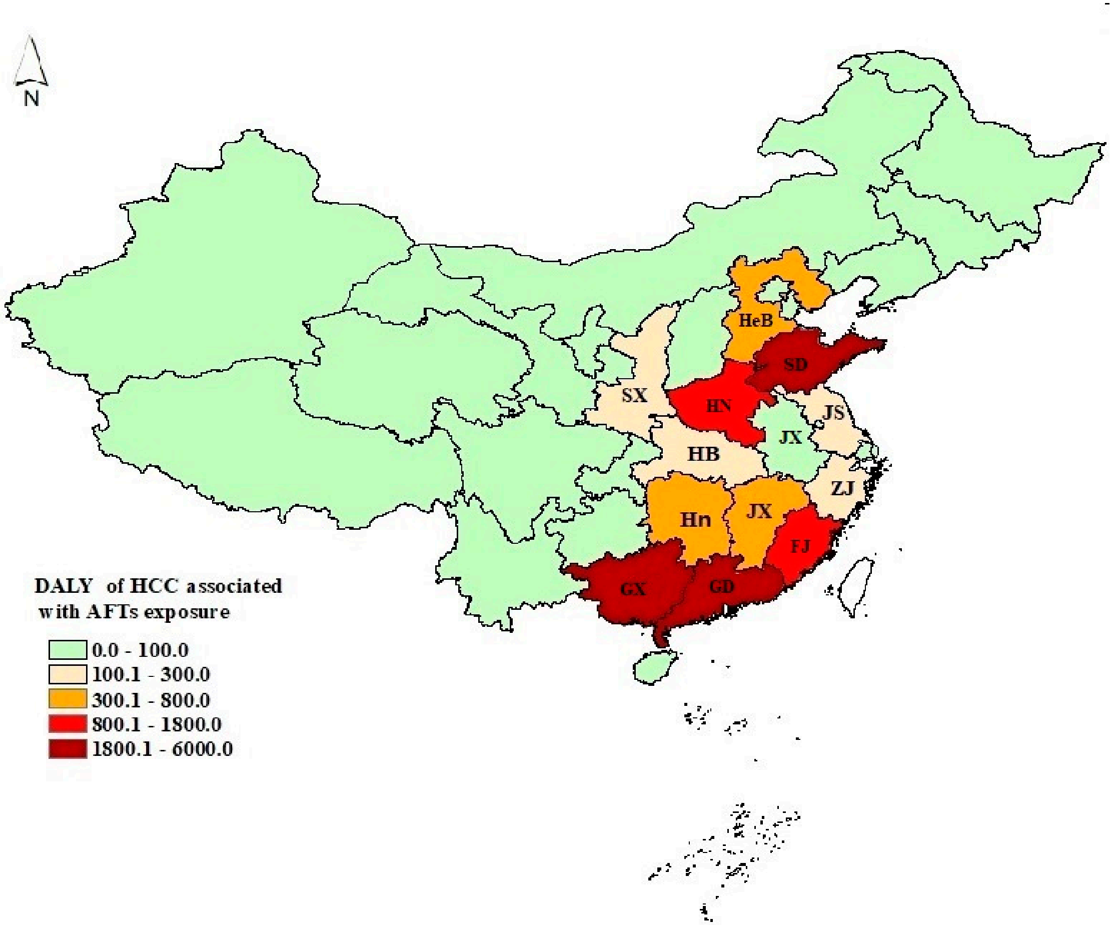

Figure S5. DALY of HCC associated with AFTs exposure from peanuts, peanut oil, corn, and corn products. HeB: Hebei, HB: Hubei, SD: Shandong, SX: Shaanxi, HN: Henan; JS: Jiangsu; Hn: Hunan, JX: Jiangxi; ZJ: Zhejiang, FJ: Fujian, GD: Guangdong, GX: Guangxi,

Table S2. Relevant information of aflatoxin contaminate in peanuts.

Province	Sample Time (year)	Aflatoxin	Detection Method	Number	Positive Number	Mean(μg/kg)	weighted Means (μg/kg)	Max(μg/kg)	Literature
Anhui	2010	AFTs	UPLC-MS / MS	58	16	0.26	9.25	-	Ma et al, 2010[1]
Anhui	2010	AFB1	HPLC	82	25	4.48		-	Ding et al, 2015[2]
Anhui	2011	AFB1	HPLC	149	68	2.48		-	Ding et al, 2015[2]
Anhui	2012	AFB1	HPLC	150	32	2.94		-	Ding et al, 2015[2]
Anhui	2013	AFB1	HPLC	149	61	12.66		-	Ding et al, 2015[2]
Anhui	2014	AFB1	HPLC	79	22	43.11		-	Ding et al, 2015[2]
Anhui	2013-2018	AFTs	HPLC-MS/MS	39	8	1.1		14.49	Qin et al, 2020[3]
Beijing	2013-2018	AFTs	HPLC-MS/MS	15	3	0.71	0.71	0.73	Qin et al, 2020[3]
Chongqing	2013-2014	AFTs	UPLC	100	39	0.834	0.97	157.2	Zhang et al, 2016[4]
Chongqing	2013	AFB1	ELISA	16	16	1.74		4.04	Feng et al, 2014[5]
Chongqing	2013-2018	AFTs	HPLC-MS/MS	20	0	1.01		1.4	Qin et al, 2020[3]
Fujian	2010-2011	AFTs	HPLC-photochemical derivatization	62	19	39.025	14.02	815	Qiu et al, 2012[6]
Fujian	2014-2015	AFB1	HPLC-fluorometer	173	>0	6.76		332.28	Liu and Pan, 2016[7]
Fujian	2010	AFTs	UPLC-MS / MS	27	7	1.15		3.7	Ma et al, 2010[1]
Fujian	2013-2018	AFTs	HPLC-MS/MS	22	5	16.49		356.7	Qin et al, 2020[3]
Gansu	2010	AFTs	UPLC-MS / MS	30	6	0.46	0.59	-	Ma et al, 2010[1]
Gansu	2013-2018	AFTs	HPLC-MS/MS	33	0	0.7		3	Qin et al, 2020[3]
Guangdong	2015-2017	AFTs	UPLC	92	40	0.44	11.73	15.816	Li et al, 2018[8]

Guangdong	2013-2018	AFTs	HPLC-MS/MS	74	13	5.13		291.5	Qin et al, 2020[3]
Guangdong	2015	AFB1	HPLC	136	32	22.95		862	Pan, 2016[9]
Guangxi	2010	AFTs	UPLC-MS / MS	34	26	12.75	7.78	107.9	Ma et al, 2010[1]
Guangxi	2013-2018	AFTs	HPLC-MS/MS	22	0	0.09		0.09	Qin et al, 2020[3]
Guizhou	2013-2018	AFTs	HPLC-MS/MS	23	1	0.21	0.21	0.59	Qin et al, 2020[3]
Hainan	2010	AFTs	UPLC-MS / MS	6	0	0	0.47		Ma et al, 2010[1]
Hainan	2013-2018	AFTs	HPLC-MS/MS	10	1	0.16		0.4	Qin et al, 2020[3]
Hainan	2019	AFB1	HPLC-MS/MS	30	4	0.67		10.27	Wang et al, 2019[10]
Hebei	2017	AFTs	HPLC-fluorometer	10	10	67.72	21.42		Cheng et al. 2018[11]
Hebei	2013-2018	AFTs	HPLC-MS/MS	30	8	5.99		67.8	Qin et al, 2020[3]
Heilongjiang	2013-2018	AFTs	HPLC-MS/MS	10	2	0.41	0.41	2.92	Qin et al, 2020[3]
Henan	2014-2015	AFTs	HPLC-fluorometer	30	18	4.57	5.89	16.1	Li et al, 2017[12]
Henan	2017	AFTs	HPLC-fluorometer	10	5	27.69			Cheng et al. 2018[11]
Henan	2013-2018	AFTs	HPLC-MS/MS	67	21	3.22		16.18	Qin et al, 2020[3]
Hubei	2010	AFTs	UPLC-MS / MS	29	20	20.43	6.55	339.6	Ma et al, 2010[1]
Hubei	2010	AFB1	HPLC	93	63	3.18		4.29	Ding et al, 2015[2]
Hubei	2011	AFB1	HPLC	99	31	2.98		0.64	Ding et al, 2015[2]

Hubei	2012	AFB1	HPLC	47	10	2.33		7.96	Ding et al, 2015[2]
Hubei	2013	AFB1	HPLC	98	33	14.86		73.23	Ding et al, 2015[2]
Hubei	2014	AFB1	HPLC	17	0	0		0	Ding et al, 2015[2]
Hubei	2013-2018	AFTs	HPLC-MS/MS	41	14	0.72		7.92	Qin et al, 2020[3]
Hunan	2010	AFTs	UPLC-MS/MS	15	4	0.57	13.49	-	Ma et al, 2010[1]
Hunan	2010	AFB1	HPLC	70	30	3		2.08	Ding et al, 2015[2]
Hunan	2011	AFB1	HPLC	86	50	11.96		33.4	Ding et al, 2015[2]
Hunan	2012	AFB1	HPLC	140	33	15.49		48.56	Ding et al, 2015[2]
Hunan	2013	AFB1	HPLC	110	34	21.51		113.5	Ding et al, 2015[2]
Hunan	2014	AFB1	HPLC	7	0	0		0	Ding et al, 2015[2]
Hunan	2013-2018	AFTs	HPLC-MS/MS	40	9	13.28	3.33	251.87	Qin et al, 2020[3]
Jiangsu	2010	AFB1	HPLC	60	0	0		0	Ding et al, 2015[2]
Jiangsu	2011	AFB1	HPLC	100	55	0.56		0.39	Ding et al, 2015[2]
Jiangsu	2012	AFB1	HPLC	100	14	0.44		0.2	Ding et al, 2015[2]
Jiangsu	2013	AFB1	HPLC	100	53	12.79		59.95	Ding et al, 2015[2]
Jiangsu	2014	AFB1	HPLC	12	0	0		0	Ding et al, 2015[2]
Jiangsu	2013-2018	AFTs	HPLC-MS/MS	49	2	0.44		3.5	Qin et al, 2020[3]
Jiangxi	2010	AFTs	UPLC-MS / MS	23	7	28.82	10.81	166.8	Ma et al, 2010[1]
Jiangxi	2010	AFB1	HPLC	92	37	6.59		12.43	Ding et al, 2015[2]
Jiangxi	2011	AFB1	HPLC	93	54	11.41		39.35	Ding et al, 2015[2]
Jiangxi	2012	AFB1	HPLC	130	44	6.49		21.76	Ding et al, 2015[2]
Jiangxi	2013	AFB1	HPLC	100	20	11.73		46.56	Ding et al, 2015[2]
Jiangxi	2014	AFB1	HPLC	24	2	7.98		0	Ding et al, 2015[2]
Jiangxi	2013-2018	AFTs	HPLC-MS/MS	20	4	33.7		343.15	Qin et al, 2020[3]
Jilin	2013-2018	AFTs	HPLC-MS/MS	10	3	1.7	1.28	1.93	Qin et al, 2020[3]
Jilin	2017	AFTs	HPLC-fluorometer	15	0	1		1	Cheng et al. 2018[11]

Liaoning	2017	AFTs	HPLC-fluorometer	10	0	1	0.34	1	Cheng et al. 2018[11]
Liaoning	2019-2020	AFTs	HPLC-MS/MS	120	13	0.28		6.616	Wang et al, 2020[13]
Neimongol	2017	AFTs	HPLC-fluorometer	5	0	1	0.34	1	Cheng et al. 2018[11]
Neimongol	2013-2018	AFTs	HPLC-MS/MS	10	0	0.01		0.01	Qin et al, 2020[3]
Ningxia	2013-2018	AFTs	HPLC-MS/MS	10	5	0.89	0.89	4.59	Qin et al, 2020[3]
Qinghai	2013-2018	AFTs	HPLC-MS/MS	14	0	1.2	1.2	4	Qin et al, 2020[3]
Shaanxi	2012-2015	AFTs	UPLC	37	9	34	34.50	323	Hu et al, 2016[14]
Shaanxi	2013-2018	AFTs	HPLC-MS/MS	27	8	35.18		316.17	Qin et al, 2020[3]
Shandong	2017	AFTs	HPLC-fluorometer	110	17	2.95	1.95		Cheng et al, 2018[11]
Shandong	2013-2018	AFTs	HPLC-MS/MS	91	1	0.74		11.4	Qin et al, 2020[3]
Shanghai	2010	AFTs	UPLC-MS/MS	54	22	18.19	12.09	334.7	Ma et al, 2010[1]
Shanghai	2013-2018	AFTs	HPLC-MS/MS	43	1	4.43		18.44	Qin et al, 2020[3]
Shanxi	2013-2018	AFTs	HPLC-MS/MS	16	0	4	4.00	4	Qin et al, 2020[3]
Sichuan	2017	AFTs	HPLC	24	4	0.3	5.75	-	Zhang et al, 2019[15]
Sichuan	2010	AFB1	HPLC	80	2	0.3		0	Ding et al, 2015[2]
Sichuan	2011	AFB1	HPLC	97	21	8.37		4.82	Ding et al, 2015[2]
Sichuan	2012	AFB1	HPLC	140	26	8.19		22.63	Ding et al, 2015[2]
Sichuan	2013	AFB1	HPLC	140	21	7.41		8.59	Ding et al, 2015[2]
Sichuan	2013-2018	AFTs	HPLC-MS/MS	54	3	0.93		8.31	Qin et al, 2020[3]
Tianjin	2013-2018	AFTs	HPLC-MS/MS	19	0	2.17	7.47	4	Qin et al, 2020[3]
Tianjin	2016-2018	AFB1	UPLC-MS/MS	200	46	7.97		189	Wu et al, 2019[16]
Xinjiang	2013-2018	AFTs	HPLC-MS/MS	40	0	0.11	0.11	0.13	Qin et al, 2020[3]
Xizang	2017	AFTs	HPLC	23	0	0	0.00	-	Zhang et al, 2019[15]
Yunan	2017	AFTs	HPLC	24	1	0.25	0.56	-	Zhang et al, 2019[15]
Yunnan	2010	AFTs	UPLC-MS / MS	16	10	0.27		-	Ma et al, 2010[1]
Yunnan	2013-2018	AFTs	HPLC-MS/MS	30	0	0.97		2	Qin et al, 2020[3]
Zhejiang	2013-2018	AFTs	HPLC-MS/MS	50	9	9.9	9.9	249.17	Qin et al, 2020[3]

AFTs: includes four aflatoxins: AFB1, AFB2, AFG1, AFG2.

Table S3. Relevant information of aflatoxin contaminate in peanut oil.

Province	Sample Time (year)	Aflatoxin	Detection Method	Number	Positive Number	Mean(μg/kg)	weighted Means (μg/kg)	Max(μg/kg)	Literature
Chongqing	2013	AFB1	ELISA	16	16	0.29	0.99	0.51	Feng et al, 2014[5]
Chongqing	2013-2018	AFTs	HPLC-MS/MS	27	0	1.4		1.40	Qin et al, 2020[3]
Chongqing	2013-2014	AFTs	HPLC-photochemical derivatization	30	12	0.13			Zhang et al, 2016[4]
Fujian	2010-2011	AFTs	HPLC-photochemical derivatization	20	9	2.962	27.42	11.4	Qiu et al, 2012[6]
Fujian	2013-2018	AFTs	HPLC-MS/MS	146	96	30.77		583.09	Qin et al, 2020[3]
Guangdong	2016-2017	AFB1	ELISA and UPLC-MS/MS	427	80	4.3881	14.42	234.8	Qi et al, 2019[17]
Guangdong	2017	AFB1	HPLC-photochemical derivatization	3221	1833	12.9		950	Song et al, 2019[18]
Guangdong	2012-2013	AFTs	HPLC-photochemical derivatization	30	-	13.21		75.3	Li et al, 2014[19]
Guangdong	2017	AFB1	HPLC	126	111	16.2		288	Yin et al, 2017[20]
Guangdong	2012-2018	AFB1	ELISA	248	214	12.83		181	Chen et al, 2017[21]
Guangdong	2019	AFB1	ELISA	243	53	21.3		114	Huang et al, 2019[22]

Guangdong	2015-2017	AFB1	HPLC	96	74	38.74		283	Zhang et al, 2020[23]
Guangdong	2013-2018	AFTs	HPLC-MS/MS	153	113	37.63		1043.50	Qin et al, 2020[3]
Guangdong	2015	AFB1	HPLC	200	110	26.69		119.7	Pan, 2016[9]
Guangdong	2015-2017	AFTs	UPLC	53	35	2.69		30.8	Li et al, 2018[8]
Guangxi	2017	AFB1	HPLC-fluorometer	146	114	30.8	31.92	320	Cheng et al, 2017[24]
Guangxi	2020	AFB1	HPLC-photochemical derivatization	183	108	16.91		243	Liang et al, 2020[25]
Guangxi	2014-2017	AFB1		310	231	16.76		510.01	Wang et al, 2020[26]
Guangxi	2014	AFB1	HPLC-fluorometer	39	>18	70.2		139.8	Liu et al, 2015[27]
Guangxi	2014	AFB1	HPLC-fluorometer	43	>16	160.3		320	Liu et al, 2015[27]
Guangxi	2014	AFB1	HPLC-fluorometer	47	>12	74.9		149.4	Liu et al, 2015[27]
Guangxi	2013-2018	AFTs	HPLC-MS/MS	76	39	13.18		149.50	Qin et al, 2020[3]
Guizhou	2013-2018	AFTs	HPLC-MS/MS	17	11	1.02	1.02	3.37	Qin et al, 2020[3]
Hebei	2013-2018	AFTs	HPLC-MS/MS	82	35	7.08	7.08	200.25	Qin et al, 2020[3]

Henan	2014-2015	AFTs	HPLC-fluorometer	87	14	1.3	1.99	13.2	Li et al, 2017[28]
Henan	2013-2018	AFTs	HPLC-MS/MS	183	29	2.32		21.32	Qin et al, 2020[3]
Hubei	2013-2018	AFTs	HPLC-MS/MS	67	2	1.60	1.60	2.00	Qin et al, 2020[3]
Jiangsu	2013-2018	AFTs	HPLC-MS/MS	33	8	1.31	1.31	4.00	Qin et al, 2020[3]
Jiangxi	2013-2018	AFTs	HPLC-MS/MS	38	12	10.29	10.29	32.99	Qin et al, 2020[3]
Jilin	2013-2018	AFTs	HPLC-MS/MS	13	0	0.5	0.50	0.50	Qin et al, 2020[3]
Liaoning	2013-2018	AFTs	HPLC-MS/MS	50	2	1.68	1.68	4.23	Qin et al, 2020[3]
Shaanxi	2012-2015	AFTs	UPLC	61	41	1.2	1.14	5. 84	Hu et al, 2016[14]
Shaanxi	2014	AFTs	HPLC-MS/MS	7	7	1.15		3.24	Li and Ma, 2016[29]
Shaanxi	2013-2018	AFTs	HPLC-MS/MS	55	34	1.08		5.64	Qin et al, 2020[3]
Shandong	2013-2018	AFTs	HPLC-MS/MS	326	103	6.78	6.78	317.20	Qin et al, 2020[3]
Yunnan	2010	AFB1	ELISA	5	5	6.819	3.44	10.027	Lan et al, 2010[30]
Yunnan	2013-2018	AFTs	HPLC-MS/MS	23	1	2.71		4.21	Qin et al, 2020[3]
Zhejiang	2016	AFB1	HPLC	345	18	0.2561	0.26	4.9	Zhou et al, 2017[31]
Zhejiang	2013-2018	AFTs	HPLC-MS/MS	18	13	0.35		1.09	Qin et al, 2020[3]

AFTs: includes four aflatoxins: AFB1, AFB2, AFG1, AFG2.

Table S4. Relevant information of aflatoxin contaminate in corn and corn product.

Province	Sample Time (year)	Aflatoxin	Detection Method	Number	Positive Number	Mean(μg/kg)	weighted Means (μg/kg)	Max(μg/kg)	Literature
Anhui	2010	AFTs	UPLC-MS / MS	30	15	1.06	1.06	1.70	Ma et al, 2010 [30]
Chongqing	2013	AFB1	ELISA	16	16	6.44	6.44	63.33	Feng et al, 2014[5]
Fujian	2010	AFTs	UPLC-MS / MS	16	6	0.57	0.57	0.70	Ma et al, 2010[1]
Gansu	2010	AFTs	UPLC-MS / MS	17	3	0.58	0.58	-	Ma et al, 2010[1]
Guangxi	2017-2019	AFB1	HPLC	186	>127	177.87		3089.00	Chen, 2020[32]
Guangxi	2010	AFTs	UPLC-MS / MS	44	39	24.72		581.30	Ma et al, 2010[1]
Hainan	2010	AFTs	UPLC-MS / MS	3	2	6.15	6.15	8.00	Ma et al, 2010[1]
Hebei	2015-2016	AFB1	ELISA	92	78	8.96	8.96	61.50	Ji et al, 2017[33]
Heilongjiang	2010	AFTs	UPLC-MS / MS	18	10	0.57	0.57	-	Ma et al, 2010[1]
Henan	2014	AFB1	HPLC	40	32	50.32	50.32	284.32	Wu et al, 2014[34]
Hubei	2010	AFTs	UPLC-MS / MS	20	12	3.40	3.40	9.50	Ma et al, 2010[1]
Hunan	2010	AFTs	UPLC-MS / MS	10	7	22.10	22.10	67.60	Ma et al, 2010[1]
Jiangxi	2010	AFTs	UPLC-MS / MS	11	5	0.43	0.43	-	Ma et al, 2010[1]
Shaanxi	2012-2015	AFTs	UPLC	268	31	1.77	1.54	16.60	Hu et al, 2016[14]
Shaanxi	2016	AFTs	HPLC	40	0	0.02		-	Hu et al, 2017[14]
Shandong	2016	AFTs	HPLC	90	8	0.14		1.69	Yang et al, 2018[35]
Shandong	2013-2014	AFTs	UPLC-Q-TOF	540	94	7.62		573.13	Wang et al, 2016[36]

Shandong	2016	AFB1	ELISA	59	57	2.11	-	10.76	Zhang et al, 2017[37]
Shanghai	2010	AFTs	UPLC-MS / MS	19	7	1.26	1.26	2.20	Ma et al, 2010[1]
Shanxi	2010	AFTs	UPLC-MS / MS	13	8	1.83	1.83	6.50	Ma et al, 2010 [1]
Tianjin	2016-2018	AFB1	UPLC-MS/MS	200	67	8.98	8.98	206.00	Wu et al, 2019[16]
Yunnan	2010	AFTs	UPLC-MS / MS	14	11	65.74	65.74	451.80	Ma et al, 2010[1]
Chongqing	2013-2014	AFTs	UPLC	63	24	0.95	0.95	29.5	Zhang et al, 2016[4]
Guangdong	2015-2017	AFTs	UPLC	90	39	0.51	0.26	4. 413	Li et al, 2018[8]
Guangdong	2012-2013	AFTs	HPLC	30	>0	0.19		0.47	Li et al, 2014[19]
Guangdong	2017	AFB1	HPLC	46	0	0.50		<0.5	Yin et al, 2017[20]
Guangdong	2012-2018	AFB1	ELISA	43	6	0.26		1	Chen et al, 2017[21]
Guangdong	2015-2017	AFB1	HPLC	339	3	0.17		6.3	Zhang et al, 2020[23]
Guangxi	2014-2017	AFB1	HPLC	58	30	3.03	3.03	43.6	Wang et al, 2020[26]
Henan	2014-2015	AFTs	HPLC-fluorometer	25	0	0.97	0.81	-	Li et al, 2017[12]

Henan	2014-2015	AFTs	HPLC-fluorometer	87	6	0.61		22	Li et al, 2017[12]
Henan	2013-2014	AFB1	HPLC	447	>0	0.48		1.81	Li et al, 2016[28]
Henan	2018-2019	AFB1	UPLC-MS/MS	100	55	2.44		140.3	Li et al, 2020[38]
Jiangsu	2011	AFTs	HPLC	81	41	10.51198	8.06	374.2	Cai et al, 2013[39]
Jiangsu	2013	AFTs	HPLC	25	8	0.12		0.66	Zhao et al, 2015[40]
Shaanxi	2012-2015	AFTs	UPLC	44	0	0.07	0.37	-	Hu et al, 2016[14]
Shaanxi	2013-2016	AFTs	UPLC-MS/MS	120	6	0.60		16.6	Hu et al, 2017[41]
Shaanxi	2016	AFTs	HPLC	40	5	0.07		0.59	Hu et al, 2017[42]
Shaanxi	2016	AFTs	HPLC	40	1	0.02		0.17	Hu et al, 2017[42]
Shaanxi	2014	AFTs	HPLC-MS/MS	10	6	1.62		6.09	Li and Ma, 2016[29]
Shandong	2016	AFTs	UPLC-MS/MS	90	29	0.23	1.44	8.04	Jiang et al, 2019[43]
Shandong	2015	AFTs	HPLC-fluorometer	40	18	3.70		17.7	Wang and Ma,2015[44]
Shandong	2014-2017	AFTs	HPLC	91	>0	0.15		3.44	Gong et al, 2018[45]
Shandong	2011-2013	AFTs	UPLC	150	78	2.352		8.54	Yang et al, 2016[46]
Yunnan	2012-2017	AFB1	UPLC-MS/MS	292	>6	1.83	1.83	2.239	Hu et al, 2020[47]
Zhejiang	2016	AFB1	HPLC	125	1	0.0744	0.07	-	Zhou et al, 2017[31]

AFTs: includes four aflatoxins: AFB1, AFB2, AFG1, AFG2.

References

1. Ma, J.; Shao B.; Lin, X.; Yu, H.; Li, F. Study on the natural occurrence of multi-mycotoxin in cereal and cereal-based product samples collected from parts of China in 2010. *Chin. J. Food Hyg*. **2011**, *23*, 481–488.
2. Ding, X.; Wu, L.; Li, P.; Zhang, Z.; Zhou, H.; Bai, Y.; Chen, X.; Jiang, J.; Risk Assessment on Dietary Exposure to Aflatoxin B(1) in Post-Harvest Peanuts in the Yangtze River Ecological Region. *Toxins* **2015**, *7*, 4157–4174.
3. Qin, M.; Liang, J.; Yang, D.; Yang, X.; Cao, P.; Wang, X.; Ma, N.; Zhang, L. Spatial analysis of dietary exposure of aflatoxins in peanuts and peanut oil in different areas of China. *Food Res. Int*. **2021**, *140*, 109899.
4. Zhang, X.; Ding, J.; Li, S.; Cheng, Y. Aflatoxin contamination in the marketed food in Wanzhou District, Chongqing City, 2013-2014. *Practical Preventive Medicine* **2016**, *23*, 426–428.
5. Feng, L.; Yang, D.; Li, H., Tian, Y., Zhao, T., Zhang, R. Survey on pollution of aflatoxin b1 in foodstuffs. *Parasitoses Infec. Dis*. **2014**, *3*, 117–119.
6. Qiu, W., Fu, W. Contamination of aflatoxins in peanuts and peanut products from Fujian. *Chin. J. Health Laboratory Technol*. **2012**, *22*, 2446–2448.
7. Liu, W.; Pan, W. Peanut aflatoxins contamination and evaluation on dietary exposure in Fujian province. *Cereal & Feed Industry* **2016**, *11*, 28–32.
8. Li, K.; Chen, W.; Qiu, F.; Liang, Z.; Yang, M; Wang, Z. Dietary exposure assessment to aflatoxins in nine kinds of foodstuff by Mont Carlo non-parametric probability approach in Shenzhen City. *Wei Sheng Yan Jiu= Journal of Hygiene Research* **2018**, *47*, 827–832.
9. Pan, Z. Hazard Analysis and Detection Method Establishment of Aflatoxin B1 in Peanut Oil and Raw Materials. Master◈ Thesis, South China Agricultural University: Guangzhou; 2016.
10. Wang, Y.; Liu, Q.; Wu, X.; Huang, X.; Wang, F.; Li, X. Determination of anatoxin B1 in peanut by high performance liquid chromatOgraphy-tandem mass spectrometry. *Journal of Food Safety and Quality* **2019**, *10*, 2059–2063.
11. Cheng, G.; Wang, S.; Hu, D.; Pang, G.; Gao, L.; Zhan, Z. Field Investigation on Aflatoxin Contamination of Peanut in Main Peanut Production Districts in 2017. *Journal of Peanut Science* **2018**, *47*, 30–33.
12. Li, S.; Yuan, P.; Fu, P.Y.; Yang, L.; Zhang, R.; Zhang. S. Investigation of Contamination Situation of Fungaltoxin in Some Food in Henan Province from 2014 to 2015. *China Health Industry* **2017**, *27*, 144–147.
13. Wang, Y.; Wang, J.; Lin, Q.; Li, G.; Guo, C.; Wu, X.; Li, H.; Ren, Z.; Chen, F. Investigation and Analysis of Aflatoxin in Peanut in Liaoning Area. *Liaoning Agricultural Sciences* **2020**, *2*, 77–79.
14. Hu, J.; Tian, L.; Wang, C.; Qiao, H.; Wang, M. Analysis on contamination of aflatoxins in food samples in Shaanxi Province from 2012–2015. *Journal of Hygiene Research* **2016**, *45*, 762–765.
15. Zhang X., Yue, X.; Ding, X.; Li, P.; Yu, Q.; Xie, H.; Zhang, Q.; Zhang, Z.; Zhang, W. Distribution and aflatoxin contamination by Aspergillus flavus in peanut from the southwest China. *Chinese Journal of Oil Crop Sciences* **2019**, *41*, 773–780.
16. Wu, X.; Zhao, K.; Lin, X. Pollution condition of mycotoxin in grain and oil in Tianjin area. *Occup and Health*. **2019**, *35*, 2996–2998.
17. Qi, N.; Yu, H.; Yang, C.; Gong, X.; Liu, Y.; Zhu, Y. Aflatoxin B1 in peanut oil from Western Guangdong, China, during 2016–2017. *Food Addit. Contam. Part B Surveill*. **2019**, *12*, 45–51.
18. Song, M.; Le, Li; Luo, Y.; Xie, C.; Chen, Z. Dietary exposure and risk assessment of aflatoxin B1 in peanut oil produced by individual workshop in Guangdong. *China Oils and Fats* **2019**, *44*, 96–101.
19. Li, K.; Qiu, F.; Jiang, L.; Yang, M. Dietary exposure assessment of aflatoxin of foodstuff and edible oil from Shenzhen residents. *Journal of Hygiene Research* **2014**, *43*, 630–636.
20. Yin, G.; Liu, S.; Liao, L. Analysis on Aflatoxin B1 Contamination in Vegetable Oils. *J. Prev. Med. Inf.* **2017**, *33*, 593–596.
21. Chen, X.; Cai, C.; Chen, Y.; He, B.; Guo, Y.; He, L.; Lu, L.; Wen, Y. Dietary exposure risk assessment of aflatoxin B1 in edible vegetable oil of residents based on margin of exposure and digital method. *Journal of Food Safety and Quality*. **2017**, *33*, 593–596.
22. Huang, S.; Luo, H.; Zeng, X.; Xiang, J.; Zhong, X.; Liu, Q. Risk assessment of aflatoxin B1 exposure in peanut oil from small workshop in Qingyuan city. *Analysis and Detection* **2019**, *21*, 66–67.
23. Zhang, W.; Liu, Y.; Liang, B.; Zhang, Y.; Zhong, X.; Luo, X.; Huang, J.; Wang, Y.; Cheng, W.; Chen, K. Probabilistic risk assessment of dietary exposure to aflatoxin B1 in Guangzhou, China. *Sci. Rep*. **2020**, *10*, 7973.
24. Cheng, H.; Zhong, X.; Chen, J.; Meng, H.; Liao, Y.; Chen, H.; Jiang, Y.; Xie, Y.; Su, Y.; Liu, Z. Exposure risk assessment of aflatoxin B1 in edible vegetable oil by using the margin of exposure in Guangxi. *Chinese Journal of Food Hygiene* **2017**, *29*, 496–499.

25. Liang, X.; Chen, F.; Li, Q.; Zhang, X.; Mo, L. Dietary exposure and risk assessment of aflatoxin B1 in the peanut oil of small workshops in Yulin City. *China Oils and Fats* **2022**, *47*, 131–136.
26. Wang, C.; Fan, Y.; Long, X.; Zhang, J.; Huang, S.; Lv, Z.; Shi, X.; Wei, Y. Exposure risk assessment of aflatoxin B1 in some foods in Nanning. *Modern Preventive Medicine* **2020**, *47*, 252–255.
27. Liu, Z.; Tang, Z.; Zhong, Y.; Cheng, H.; Meng, H.; Jiang, Y.; Chen, H.; Xie, Y.; Su, Y.; Yao, X. Investigation of aflatoxin B1 level in edible vegetable oil in the urban and rural areas of Guangxi in 2014. *J. Applied. Prev. Med.* **2015**, *21*, 377–380.
28. Li, S.; Yang, L.; Yuan, P.; Fu, P.; Zhou, S.; Chao, F.; Zhang, S.; Zhang, D. Assessment of aflatoxin B1 dietary exposure among Henan residents. *Modern Preventive Medicine* **2016**, *43*, 1008–1010.
29. Li, Y.; Ma, Y. Analysis of mycotoxin pollution in some foods in Xianyang city in 2014. *Chin. J. Health. Lab. Tec.* **2016**, *26*, 570–572.
30. Lan, S.; Liu, H.; Mei, W.; Yang, F. Determination of aflatoxin B1 in edible vegetable oil by enzymelinked immunosorbent assay. *Journal of Cereals & Oils* **2010**, *4*, 39–41.
31. Zhou, Z.; Xing, J.; Ying, L.; Zhou, X.; Zhang, S.; Li, X.; Shen, J. Investigation and analysis of aflatoxin B1 in edible vegetable oil. *China Oils and Fats* **2017**, *42*, 66–69.
32. Chen, J. Investigation and analysis on mycotoxin pollution of maize in Baise city from 2017 to 2019. *Journal of Food Safety and Quality* **2020**, *11*, 4029–4033.
33. Ji, H.; Li, Y.; Wen, Z.; Liu, Y.; Li, X.; Li, H. Aflatoxin B1 contamination detection and safety evaluation for corn at storage in Hubei procince. Cereal & Feed Industry 2017, 3, 19–21.
34. Wu, R.; Li, F.; Zhang, K. Aflatoxin B1 Contamination Detection and Safety Evaluation for Corn in Nanyang. *Journal of Anhui Agri.* **2014**, *42*, 10306–10336.
35. Yang, X.; Liu, S.; Cao, X.; Liu, Z.; Jiao, H. Monitoring results of mycotoxin in 90 fresh corns in Shandong in 2016. *Occup. and Health* **2018**, *34*, 188–191.
36. Wang, Y.; Dong, Y.; Yue, H.; Li, Z.; Chen, Y.; Wang, Y.; Deng, L.; Zhao, S. Investigatin and analysis on mycotoxins contamination of maize in Shandong province. *Oils and Foods* **2016**, *24*, 69–73.
37. Zhang, H.; Li, F.; Lv, S.; Tang, S.; Yu, J. Investigation report on mycotoxin contamination of 2016 autumn harvest corn in Zouping County. *Animal Health in China* **2017**, *19*, 1–3.
38. Li, J.; Wang, S.; Wu, J.; Shen, L.; Yao, X. Investigation of mycotoxins in grain and its products in Henan Province. *Chinese Journal of Food Hygiene* **2020**, *32*, 418–421.
39. Cai, M.; Ji, W.; Liu, L.; Ma, Y. Monitoring on total aflatoxins in commercial peanuts and corn in Jiangsu area. *Chin. J. Health Lab. Tec.* **2013**, *23*, 2504–2505.
40. Zhao, J.; Huang, J.; Huang, W. Analysis on the chemical pollutants and harmful factors in Taizhou in 2013. *Chin. J. Health Lab. Tec.* **2015**, *25*, 1262–1265.
41. Hu, J.; Qiao, H.; Tian L.; Wang, M.; Wang, C.; Guo, R. Mycotoxin contamination of cereals and their products in Shaanxi Province from 2013 to 2016. *Journal of Hygiene Research* **2017**, *46*, 1013–1015.
42. Hu, J.; Tian, L.; Wang, M.; Wang, C.; Guo, R.; Qiao, H. Mycotoxins contamination in 120 corn products on sale, Shaanxi. *Modern Preventive Medicine* **2017**, *44*, 1593–1596.
43. Jiang, D.; Li, F.; Zheng, F.; Zhou, J.; Li, L.; Shen, F.; et al. Occurrence and dietary exposure assessment of multiple mycotoxins in corn-based food products from Shandong, China. *Food Addit. Contam. Part B Surveill*. **2019**, *12*, 10–17. doi: 10.1080/19393210.2018.1503341.
44. Wang, D.; Ma, S. Analysis of Aflatoxin contamination in some food in Weifang. *The Urban and Rural enterprise Health in China* **2015**, *30*, 71–73.
45. Gong, C.; Dong, F.; Wang, C. Investigation and Analysis of Mycotoxins Contamination in Cereal and Its Products Sold in Yantai Market. *Food Research and Development* **2018**, *39*, 189–194.
46. Yang, X.; Jiao, H.; Liu, S.; Cao, X. Aflatoxin contamination investigation of vegetable oil and corn flour samples in Jinan 2011-2013. *Chin. J. of PHM.* **2016**, *32*, 681–683.
47. Hu, W.; Dong, H.; Ning, X.; Lin, J.; Liu, Z. Risk assessment of dietary exposure to aflatoxin B1 in parts foods in Yunnan province. *Journal of Food Safety and Quality* **2020**, *11*, 5215–5219.
